# Defect Etching in Carbon Nanotube Walls for Porous Carbon Nanoreactors: Implications for CO_2_ Sorption and the Hydrosilylation of Phenylacetylene

**DOI:** 10.1021/acsanm.1c03803

**Published:** 2022-02-07

**Authors:** Maxwell A. Astle, Andreas Weilhard, Graham A. Rance, Tara M. LeMercier, Craig T. Stoppiello, Luke T. Norman, Jesum Alves Fernandes, Andrei N. Khlobystov

**Affiliations:** ^†^School of Chemistry and ^‡^Nanoscale and Microscale Research Centre (nmRC), University of Nottingham, University Park, Nottingham NG7 2RD, United Kingdom

**Keywords:** carbon nanotubes, guided etching, modulating catalysts, nanoreactors, CO_2_ sorption, hydrosilylation reaction

## Abstract

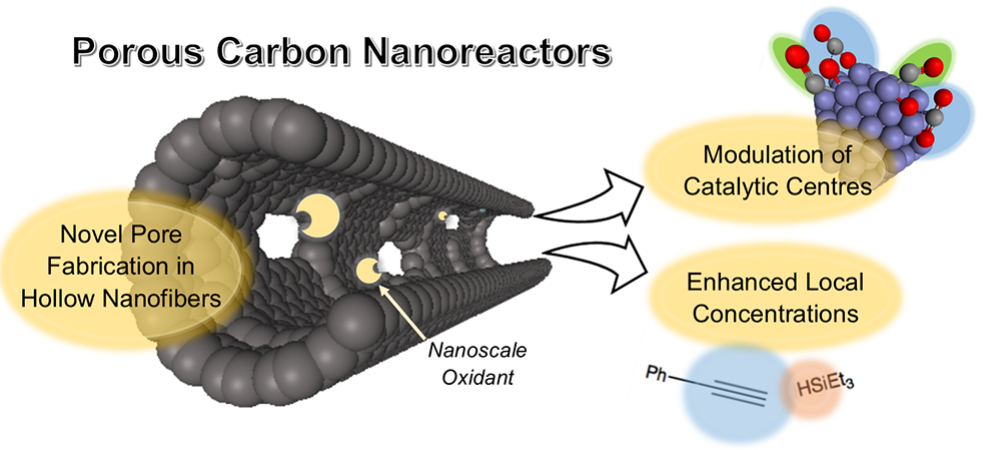

A method of pore fabrication in the walls of carbon nanotubes has been developed, leading to porous nanotubes that have been filled with catalysts and utilized in liquid- and gas-phase reactions. Chromium oxide nanoparticles have been utilized as highly effective etchants of carbon nanotube sidewalls. Tuning the thermal profile and loading of this nanoscale oxidant, both of which influence the localized oxidation of the carbon, have allowed the controlled formation of defects and holes with openings of 40–60 nm, penetrating through several layers of the graphitic carbon nanotube sidewall, resulting in templated nanopore propagation. The porous carbon nanotubes have been demonstrated as catalytic nanoreactors, effectively stabilizing catalytic nanoparticles against agglomeration and modulating the reaction environment around active centers. CO_2_ sorption on ruthenium nanoparticles (RuNPs) inside nanoreactors led to distinctive surface-bound intermediates (such as carbonate species), compared to RuNPs on amorphous carbon. Introducing pores in nanoreactors modulates the strength of absorption of these intermediates, as they bond more strongly on RuNPs in porous nanoreactors as compared to the nanoreactors without pores. In the liquid-phase hydrosilylation of phenylacetylene, the confinement of Rh_4_(CO)_12_ catalyst centers within the porous nanoreactors changes the distribution of the products relative to those observed in the absence of the additional pores. These changes have been attributed to the enhanced local concentration of phenylacetylene and the environment in which the catalytic centers reside within the porous carbon host.

## Introduction

With the application of carbon nanotubes (CNTs) rapidly expanding into several areas, the controlled formation of defects and the modulation of the surface properties are becoming increasingly important.^1,2^ One of the most innovative applications of carbon nanotubes is as nanoscale reaction vessels for catalytic reactions, where the interactions between catalytic metal nanoparticles and the host nanostructure subtly tune the reaction pathway.^3−6^ This nanoscale confinement of catalysts within nanoreactors has been shown to enhance the product selectivity and stabilize the catalyst against sintering and agglomeration.^7^ Furthermore, the ability to control the structure of the nanoreactor may offer an opportunity to reduce activation energy barriers and improve reaction kinetics in the future. The structural defects of carbon nanotubes (especially single-walled carbon nanotubes) can influence the behavior of electrons, excitons, and phonons, fundamentally controlling the electronic, optical, thermal, and mechanical properties of the carbon nanostructure.^8−11^ In contrast, the impact of defects on the chemical properties of CNTs, especially those related to catalysis, still requires systematic investigation.^12,13^ Despite recent progress, harnessing the structure and geometry of carbon nanotubes for catalysis remains a significant challenge, and there is a clear need to correlate the properties of the catalytic centers embedded within the CNT host with the nanotube structure itself.

Hollow graphitized carbon nanofibers (GNFs) are a special class of multiwalled carbon nanotubes that possess corrugated internal structures due to a stacked-cups morphology encased within concentric tubes. The 3–4 nm high folds resulting from the stacked internal structure provide ideal sites for molecular adhesion due to maximized van der Waals interactions, mimicking the spatial confinement effects observed inside narrower carbon nanotubes.^11^ Their large internal diameters result in the termini always being open, thereby allowing highly efficient transport of molecules through the internal volume. The high pyramidalization angle at the folds of the corrugated interior of the GNFs, which enhances guest–host interactions, represents a perfect environment for a catalytic nanoparticle.^14−16^ Unlike smaller diameter CNT nanoreactors, GNF nanoreactors of larger internal diameters (30–60 nm) allow for the effective transport of molecules into, through, and out of the nanoreactor.^17,18^ As a result, GNFs represent an optimal nanoscale reactor, providing effective confinement and stabilization of catalytic centers while allowing fast diffusion of reactants, and thus have shown promise in several processes.^11,17,19−23^ However, developing effective approaches for the controlled modulation of the surfaces of thick multiwalled carbon nanostructures, such as GNFs, represents a unique challenge due to their high thermal and chemical inertness and fragile stacked-cones structure supported only by van der Waals interactions.

Methods such as lithography and templated growth have been successfully applied as a defect formation strategy in several carbon nanostructures, but these methods are intrinsically unscalable which thus limits their practical applications for the fabrication of catalyst supports and nanoreactors.^24^ Solution-phase etching techniques with inorganic acids and bases are widely used for graphene-based materials but are less suitable for carbon nanotubes as this method is not toposelective, and therefore defects cannot be formed in specific locations.^22,25^ Due to the chemical robustness and stability of carbon nanotubes, guided etching with catalytic or reactive nanoparticles has grown in popularity and provides a scalable method to form porous carbon nanotubes with a certain level of control of the shape and size of the holes within the structures. Silver nanoparticles are often employed to catalyze the generation of defects in carbon nanomaterials; however, this method is costly and often uncontrollable as Ag nanoparticles have been shown to be very effective at shortening and cutting nanotubes rather than simply modulating the surface.^26,27^ The residual catalyst from nanotube synthesis (often Fe) has been reported to catalyze defect formation in nanotubes when heated in air, with the oxidation of the metal likely responsible for the destructive nature of the metal species in this case.^28^ Yet, utilizing residual metals from the carbon nanotube growth process does not provide a versatile approach for generating defects in other carbon nanostructures and also offers no control over the location or quantity of the defects in the specific examples.^28^ Oxidation in air at elevated temperature has been shown as an efficient method to assist in controllable hole growth if defects are already present in the nanotube; however, to fully exploit this, an extra step is often required where a catalyst chemically etches pristine carbon surfaces before air oxidation.^29^ Although precious metals and the residual carbon nanotube synthesis catalysts have been exploited to generate defects in carbon nanotubes, there is a need for a more sustainable, scalable, and reliable approach for controlled defect fabrication in nanotubes.

**Scheme 1:**
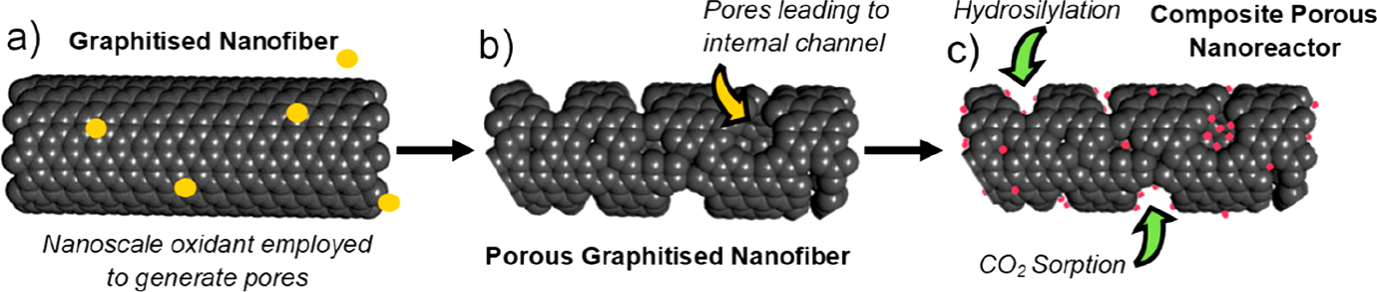
Schematic Representation of This Study An abundant nanoscale oxidant can be selectively deposited onto the surfaces of GNFs (a), connecting the exterior surface of the carbon nanoreactors with the interior, to generate porous carbon nanofibers (b). Deposition of metal nanoparticles and complexes leads to nanoreactors for CO_2_ sorption and alkyne hydrosilylation (c).

In this study, we report the use of chromium oxide nanoparticles, an abundant nanoscale oxidant that can be selectively deposited onto the surfaces of GNFs, to promote a highly controlled etching process through the 50–70 layers of carbon connecting the exterior surface of the carbon nanoreactors with the interior. Pores with openings of 40–60 nm permeate the walls of these nanoscale carbon cylinders without compromising the overall structural integrity of the nanotube, thus enabling the use of porous nanotubes as catalytic nanoreactors. Porous nanoreactors were loaded with ruthenium nanoparticles or Rh_4_(CO)_12_, and their functional properties were investigated, demonstrating their use as effective catalytic nanoreactors in CO_2_ sorption and alkyne hydrosilylation, respectively (Scheme 1).

## Results and Discussion

### Oxidative Etching of Graphitized Nanofibers

**Figure 1:**
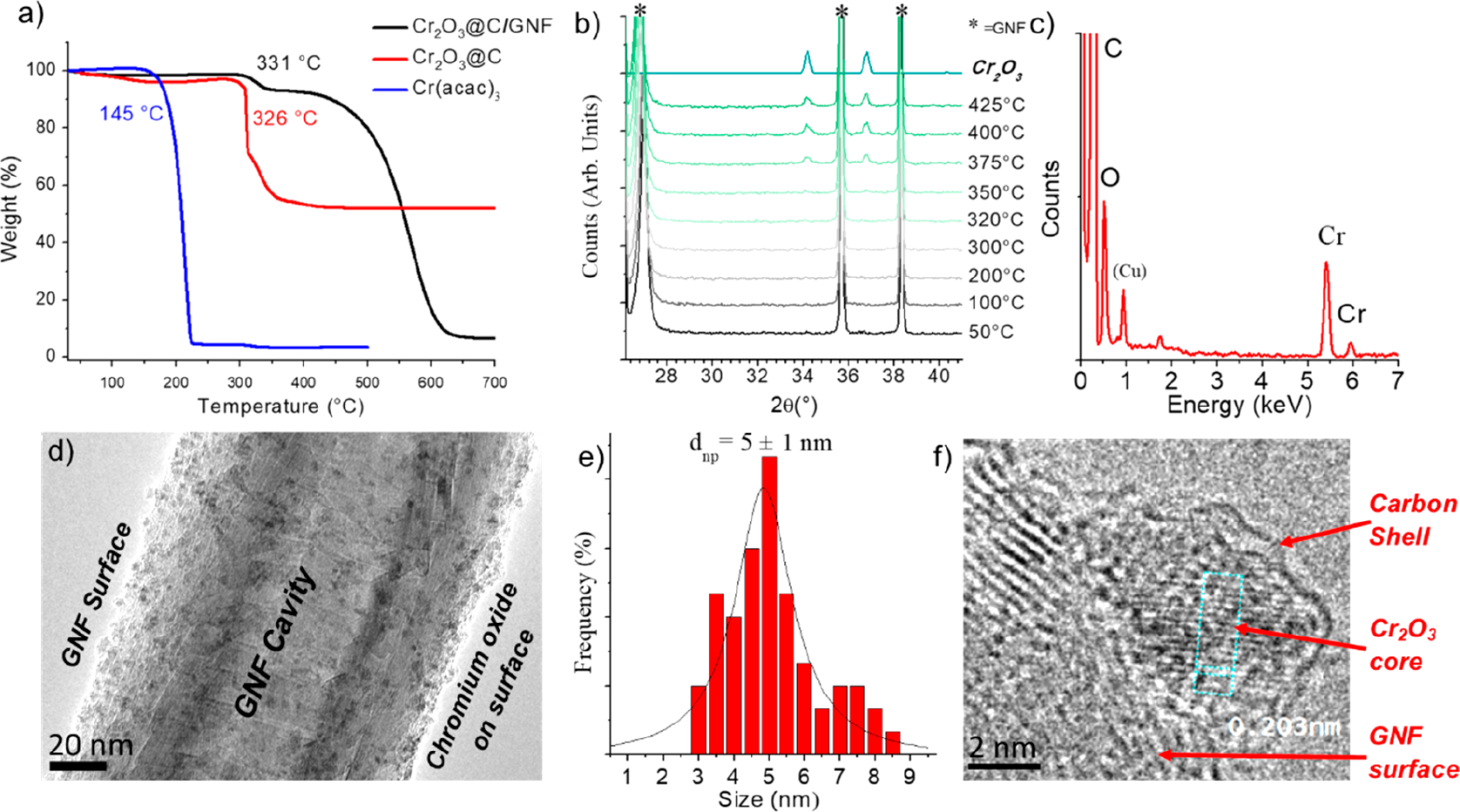
Thermograms in air of Cr(acac)_3_ with (red) and without (blue) the prior high thermal treatment in Ar, as performed during the preparation of (Cr_2_O_3_@C)/GNF, and of the composite material (Cr_2_O_3_@C)/GNF (black) (a). The weight loss at 330 °C corresponds to the oxidation of carbon shells around chromium oxide nanoparticles and above 500 °C is related to the oxidation of GNF. TGA of the GNF only shows high thermal stability up to 600 °C, with no weight loss observed below this temperature (Figure S5, Supporting Information). PXRD pattern of the (Cr_2_O_3_@C)/GNF composite material (black), with peaks at 27, 35, and 38° only, corresponding to the lattice of the GNF, due to the small size or low crystallinity of the chromium oxide nanoparticles, which become more crystalline as they transform into larger Cr_2_O_3_ nanoparticles when thermally treated in air (b). The EDX spectrum of (Cr_2_O_3_@C)/GNF confirms the presence of chromium oxide nanoparticles (the atomic ratio for chromium:oxygen was shown to be 1:4, where the excess is associated with oxygen groups on the GNF, and the Cu peak is due to the sample holder) (c). A representative TEM image of (Cr_2_O_3_@C)/GNF (d), with a nanoparticle size distribution plot (e). The HRTEM image of an individual Cr_2_O_3_@C particle adsorbed on the GNF (f), revealing lattice fringes of Cr_2_O_3_ and a carbon shell surrounding the nanoparticle.

Chromium(III) acetylacetonate (Cr(acac)_3_) has been employed as a molecular precursor for nanoparticles guiding the catalytic oxidation of GNFs. Cr(acac)_3_ was sublimed at 160 °C in vacuum and deposited onto GNF surfaces; once adsorbed, Cr(acac)_3_ was rapidly heated in a sealed vessel under Ar (inert atmosphere) to 500 °C to trigger the decomposition of the precursor and loss of the organic ligands, affording nanoparticles. Transmission electron microscopy (TEM) imaging and complementary energy dispersive X-ray spectroscopy (EDX) analysis of the resultant material confirm that small nanoparticles (4.9 ± 1.3 nm), with darker contrast than the carbon of the GNFs, are formed on the interior and exterior surfaces of the hollow nanofibers and are composed of chromium and oxygen, supporting the existence of predominantly an oxide species (Figure 1c–e). However, as the decomposition of Cr(acac)_3_ in an argon atmosphere does not provide enough oxygen to fully transform the metal to the corresponding oxide, small nanoparticles with low crystallinity are seen in high-resolution transmission electron microscopy (HRTEM) images (Figure 1f). This is consistent with powder X-ray diffraction (PXRD) analysis, where no peaks were observed in the diffractogram, indicating either a small particle size and/or low crystallinity of this phase,^30,31^ until the material was heated above 375 °C in air at which point peaks at 2θ = 24, 33, and 36°, corresponding to well-defined crystalline phases of chromium(III) oxide (Cr_2_O_3_), emerge (Figure 1b). Electron energy loss spectroscopy (EELS) and X-ray photoelectron spectroscopy (XPS) analyses of the unannealed material confirm that chromium is predominantly in the +3 oxidation state (Figures S1 and S2, Supporting Information).^32−34^

Interestingly, HRTEM additionally reveals that the chromium oxide nanoparticles adsorbed on GNFs are surrounded by an amorphous carbon shell (Figure 1f). These shells likely act as a stabilizer for the small nanoparticles, controlling their growth and preventing coalescence. As such, the composite material will be herein referred to as (Cr_2_O_3_@C)/GNF, with the carbon-coated nanoparticles crucial for the pore formation in GNFs, as demonstrated later. It is important to note that performing the decomposition step under an inert atmosphere is essential to prevent complete decomposition of the acetylacetonate ligands of the precursor complex into gaseous products and promote the formation of this carbon char around the nanoparticles (Scheme S1, Supporting Information). In a control experiment, Cr(acac)_3_ in the absence of nanotubes was treated using an identical procedure, and the decomposition product was analyzed by Raman spectroscopy and TGA. D and G bands, characteristic of a semigraphitized carbon phase, were observed in the Raman spectrum of the sample after Cr(acac)_3_ decomposition (Figure S3, Supporting Information). TGA of the decomposed precursor indicated no weight loss associated with the organic acac groups (expected around 145 °C based on thermal analysis of Cr(acac)_3_); rather, a new weight loss above 300 °C associated with amorphous carbon oxidation was noted (Figure 1a). Importantly, this weight loss is also seen in the thermogram of (Cr_2_O_3_@C)/GNF (Figure 1a) and when coupled gas analyzer mass spectrometry (MS) shows the release of CO_2_ at 336 °C when heated in air (Figure S4, Supporting Information). Oxidation of the GNF carbon in (Cr_2_O_3_@C)/GNF commences at 495 °C, which is significantly lower (by 176 °C) than that of an as-received GNF due to the catalysis of carbon oxidation by chromium oxide (Figure S5, Supporting Information). Upon complete combustion of the carbon in (Cr_2_O_3_@C)/GNF, TGA confirms a residual weight of between 2.7 and 6.5% depending on the amount of Cr(acac)_3_ precursor added to the GNF.

**Figure 2:**
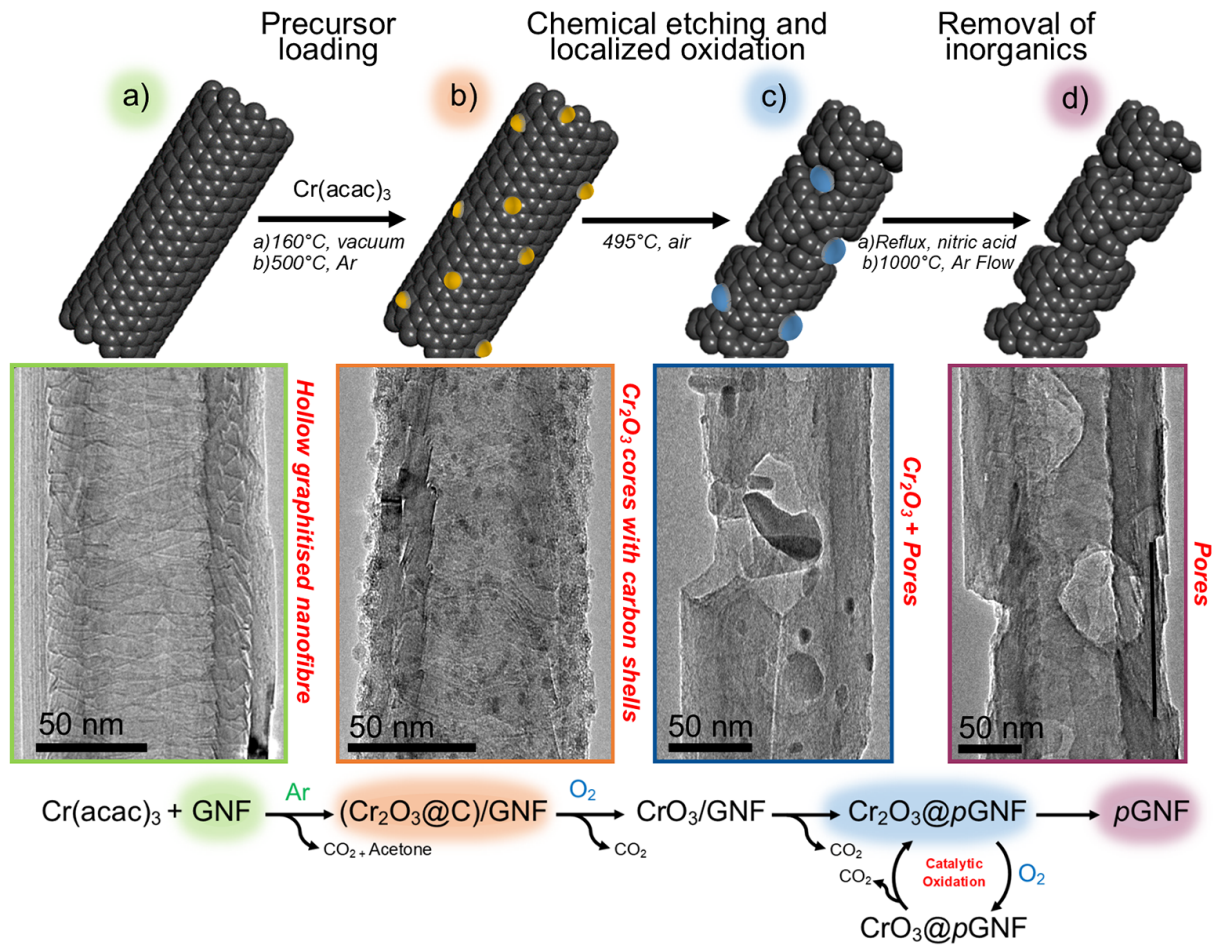
Schematic diagram with corresponding representative TEM images of the stages involved in porous carbon nanoreactor formation. GNFs (a) are exposed to vapors of a chromium acetylacetonate, followed by heating in an argon atmosphere to form chromium oxide nanoparticles attached to the GNF surface (b). Subsequent thermal treatment in air results in the localized oxidation and etching of the carbon nanotube surface promoted by the chromium oxide nanoparticles (c). Treatment at 495 °C in air determines the pore array within the nanotube and provides control over the diameter and length of the pores. Finally, refluxing the composite materials in nitric acid removes any metal oxide species, and a final thermal treatment under Ar removes oxygen groups from the carbon surface, yielding *p*GNF (d). A reaction scheme has been proposed based on TGA, MS, XRD, and TEM measurements: initially formed Cr_2_O_3_ nanoparticles on the GNF under argon ((Cr_2_O_3_@C)/GNF) are wrapped in a thin layer of carbon supplied by acetylacetonate ligands, which stabilizes the nanoparticles at ∼5 nm diameters. When heated in air up to 495 °C, first the carbon shell around Cr_2_O_3_ is removed, followed by transformation to CrO_3_ which acts as a local oxidant, converting the carbon of the GNF to CO_2_ and allowing a pore to propagate. The process is self-retarding as the reactivity of chromium oxide nanoparticles diminishes as they grow larger during the heating.

This careful thermogravimetric analysis highlights the important role that the passivated nanoparticles play in controlling defect formation in nanotubes. Below the carbon oxidation temperature, undesired nanoparticle growth is inhibited which would otherwise reduce their active surface area and consequent catalytic activity. However, once heated above 336 °C in air, the amorphous carbon shell surrounding the small catalytic nanoparticles combusts, allowing the chromium oxide to contact the surface of the graphitized carbon nanofiber. Concurrent exposure of these highly active nanoparticles to air triggers the oxidation to CrO_3_ at the surface of the nanoparticle, which is followed by the subsequent reaction of CrO_3_ with the carbon of the GNF surface. At the nanoscale, the increased number of low-coordinate Cr ions on the surface can, at elevated temperatures, be more easily reduced (by carbon) but simultaneously reoxidized (by atmospheric oxygen), thus providing a catalytic cycle removing the carbon of the GNF in the vicinity of chromium oxide nanoparticles (Figure 2).^35,36^^27^ This would, therefore, result in a localized and controllable site-selective pore formation in the GNF wall (Figure 2). This process was followed by an ex-situ TEM imaging before and after heating to 400 °C—when the carbon shells are completely lost (Figure S6, Supporting Information). These measurements show that there is a striking change in the morphology and increase in size (to 32.6 ± 14.1 nm) of the nanoparticles, which happens in conjunction with site-selective oxidation of the GNF in the immediate vicinity of the nanoparticle (Figure 2c and Figure S7, Supporting Information). It has been observed that the chromium oxide surface is able to remove carbon atoms from the GNF structure by oxidation which leads to the nanoparticles burrowing into the nanotube surface. As more carbon becomes utilized and ejected (as CO_2_) within this catalytic cycle, the nanoparticles sink further into the sidewall creating defects that can lead to fully penetrating pores with the assistance of elevated temperatures and air. Eventually the nanoparticles become deactivated due to the increase in the particle size which reduces the reactive surface area and sites which can promote the oxidation. The deactivation of the catalyst and self-termination of the oxidation process are important factors as they stop the complete destruction of the GNF and preserve its overall cylindrical morphology. The thermal profile of the assisted carbon oxidation needed to form holes that propagate through all walls of the nanotubes depends heavily on the carbon nanotube support used; a composite formed using thinner multiwalled carbon nanotubes (diameter approximately 5–8 nm) showed similar decreases in carbon oxidation temperature and evidence of hole formation but required heat treatment at 470 °C for 10 min to facilitate hole production effectively (Figures S8 and S9, Supporting Information). This suggests that nanoparticle-assisted carbon oxidation occurs at temperatures above 450 °C and indicates that this method of pore formation can be translated to other forms of carbon nanotubes and other carbon structures, with different thicknesses and degrees of graphitization.

To investigate the selectivity and potential for the control of pore formation, experiments were undertaken to optimize the site-selective oxidation of graphitized nanofibers, using TGA as an indication of defect formation and pore growth. It was shown that there was a strong positive correlation between chromium oxide loading on the GNF surface and the percentage of weight loss at the temperature of pore formation, 495 °C (Table 1, entries B–D), thus confirming the catalytic role of chromium oxide in the carbon oxidation process. At the highest loading of chromium achieved, no pores were observed before thermal treatment up to 375 °C, highlighting that the pore formation process is dependent on a combination of the loss of carbon shells (temperature above 340 °C) and assisted oxidation from the air which occurs at temperatures closer to 495 °C (Table 1, entry A). Although lower loadings were still able to produce weight losses during the etching process when heated to 495 °C, the result was an increase in deep recesses compared to pores. Moreover, it was also observed that a weight loss of around 23–30% during the pore formation step was required to consistently deliver pores that penetrate into the internal cavity of these carbon nanomaterials. The heating rate was also found to be important: ramp rates lower than 10 °C/min all appear to achieve similar results, but increasing the ramp rate leads to dramatically increased weight losses and larger defect diameters (Table 1, entries E and F). TEM analysis of this sample showed that many graphitized nanofibers were cut during the fast-thermal process and negatively impacted upon the controllability of the pore formation (Figure S10, Supporting Information). Scaled-up preparation of porous nanotubes using a tube furnace, important for later catalytic measurements, was realized, with weight losses commensurate with those achieved during small-scale processing in the furnace of the TGA instrument (Table 1, entries C and G). For bulk synthesis, a slower thermal ramp rate and consistent weight loading of the carbon oxidation catalyst were explored; however, the duration of the isothermal hold temperature was varied to deliver different levels of hole formation (Table 1, entries G–I). By increasing the isotherm in the air oxidation process, the weight loss can be tuned to ensure complete hole formation, and this strategy provides an easy way to synthesize porous nanotubes with desired specifications.

**Table 1:** Parameters and Conditions Optimized for Pore Formation in GNFs^a^

			pore formation conditions^c^				
parameters investigated	sample	chromium oxide loading (weight %)^b^	ramp rate(°C/min)	hold temperature (°C)	isotherm (min)^d^	weight loss after thermal treatment (%)	average pore diameter (nm)^e^
	A	6.5	10	375	10	7.4	N/A
weight loading of chromium oxide	B	6.5	10	495	10	28.1	49 ± 13
	C	5.7	10	495	10	24.9	58 ± 15
	D	2.7	10	495	10	10.8	39 ± 13
ramp rate conditions	E	6.5	15	495	10	38.4	77 ± 26
	F	6.5	5	495	10	29.4	48 ± 14
scale-up with varying isothermal holds	G	5.7	10	495	10	29.2	60 ± 19
	H	5.5	10	495	8	26.8	52 ± 16
	I	5.4	10	495	5	23.0	43 ± 13

a Small changes in the experimental setup between TGA and tube furnace experiments, such as variations in the gas flow, ramp rates, and temperature gradients, account for the subtle differences observed.

b Measured as residual weight by TGA after heating to 1000 °C in air.

c All measurements were performed in air. Thermal treatments for samples A–F were all performed using small batches in the furnace of the thermogravimetric analyzer which allowed for careful control of the parameters. The thermal treatments for G–I represent a scaled-up procedure which was performed in a tube furnace where gas flows and ramp rates were less controllable than in TGA.

d Once isotherms were completed, samples were cooled in an Ar atmosphere.

e The pore diameter was calculated using TEM analysis. All samples were refluxed in nitric acid for 1 h to remove the chromium oxide and thermally treated at 900 °C in Ar to remove oxygen groups formed during acid treatment.

**Figure 3:**
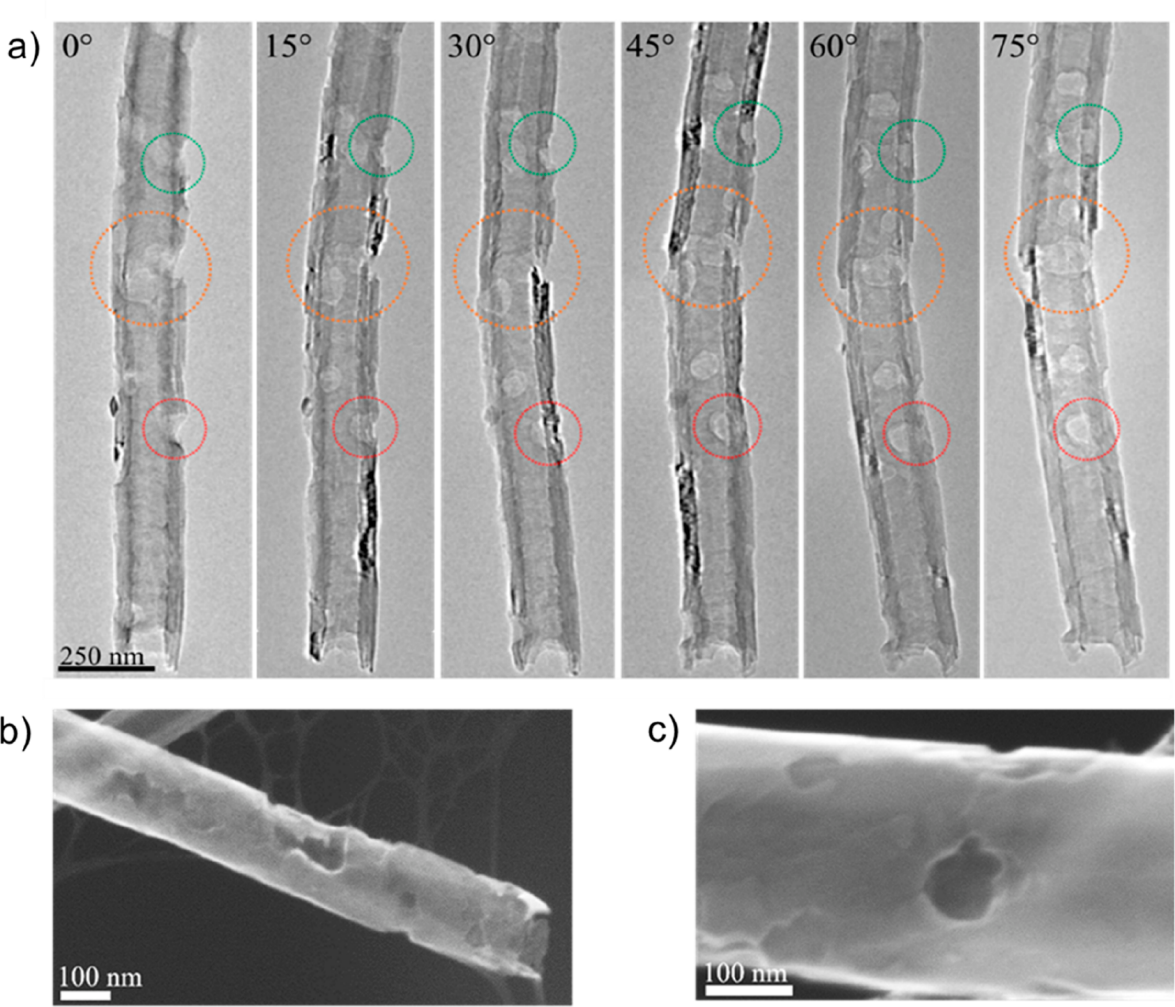
(a) TEM tilt series images of a porous graphitized nanofiber (*p*GNF), rotating around the nanotube growth axis, which allows for confirmation of effective pore formation and penetration through the whole sidewall of the carbon nanotubes. The positions highlighted by red and orange circles show examples of full pore formations, whereas the green circle indicates a surface defect that has not developed into a complete pore. (b, c) Field-emission gun scanning electron microscopy images of a *p*GNF surface.

In the final step, refluxing in concentrated nitric acid for 1 h was found to remove all the chromium oxide, while not damaging the main carbon nanotube structure (Figure 2d), and afforded porous graphitized carbon nanofibers (*p*GNFs). After the acid wash of the porous GNFs, thermal treatment at 1000 °C in Ar led to the removal of functional groups (such as carboxyls, lactones, and phenols) and resulted in reconstructed graphitic edges around the defects and restored the graphitic structure by annealing dangling bonds around the pore.^37,38^ TGA, TEM, and EDX spectroscopy were used to confirm the removal of the all the chromium oxide to ensure only pure porous nanofibers were taken forward for application, and Raman Spectroscopy highlighted the increased *I*_D_:*I*_G_ ratio after the new pores/defects were present (Figure 2d, Figures S5, S11, and S12, Supporting Information). To confirm pore propagation through the sidewall, TEM tomography (Figure 3a) and scanning electron microscopy (SEM) (Figure 3b and c and Figure S13, Supporting Information) were employed. From this analysis, the site-selective oxidation is even more pronounced, clearly resulting in the opening of nanopores into the GNF internal cavities. This method can be a useful nanofabrication tool for constructing nanoreactor systems as well as other applications of nanocarbons where control of molecular or ionic transport is required at the nanoscale.

### Porous Carbon Nanoreactors in Gas-Phase Reactions

**Figure 4:**
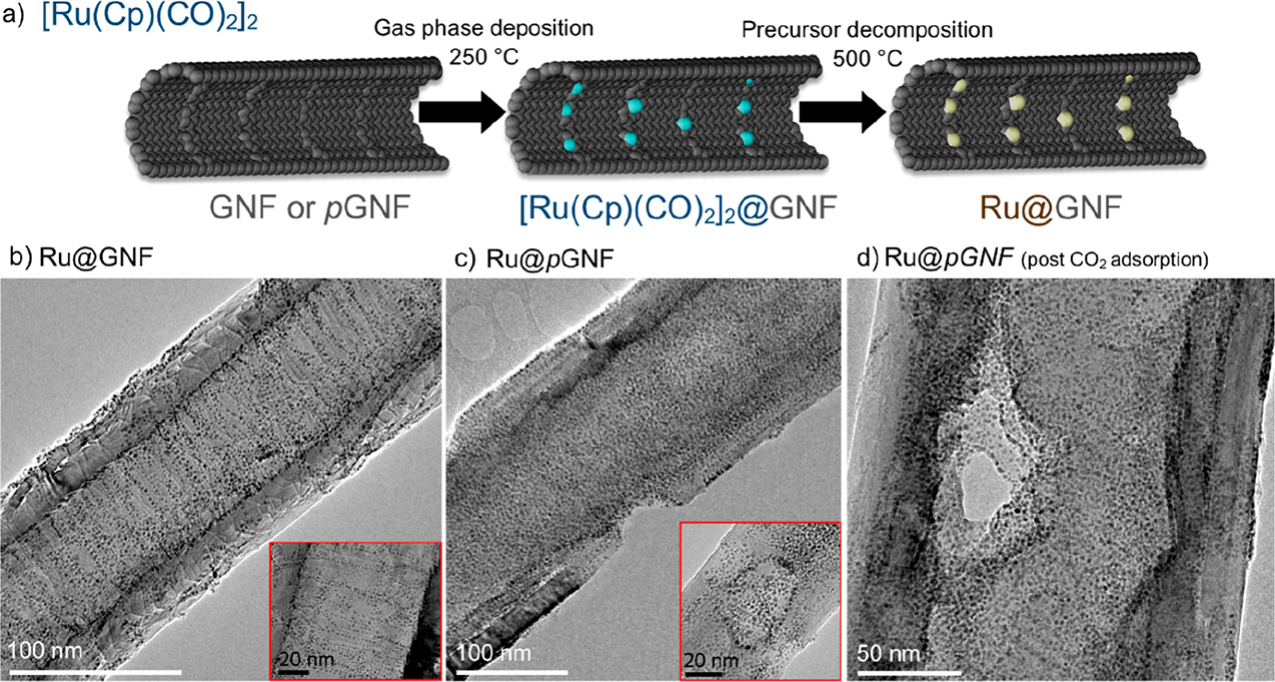
Schematic diagram illustrating the formation process for Ru nanoparticles inside carbon nanoreactors (a). Inset images highlight the favorable anchoring points within the nanofiber structures; these are the internal corrugations or defective hole periphery sites, respectively. TEM images of Ru nanoparticles within (b) as-received and (c) porous GNFs. Only a small increase in nanoparticle size for the RuNP@*p*GNF during the CO_2_ adsorption experiments between 100 and 500 °C (d) was noted. Inset images highlight the preferential anchoring points within the nanotube structures: the internal step edges (b) or pores (c) in pristine GNF and *p*GNF, respectively.

Discrete ruthenium nanoparticles (RuNPs) were confined within GNFs by sublimation of [Ru(Cp)(CO)_2_]_2_; this gas-phase filling approach allows unrestricted diffusion of precursor molecules within the nanotubes, which combined with low precursor loading results in the preferential encapsulation within the internal channel of the GNFs.^10,22^ Direct comparison between as-received and porous GNFs allowed us to probe the role of the pores in the physical properties of the confined catalysts (Figure 4), with control of the location and position of the catalytic species found to be specifically influenced. In the as-received GNFs, the internal step edges within the nanofiber stabilize small nanoparticles as they act as effective anchoring sites for nanoparticles adhesion; from TEM and EDX this was shown to lead to encapsulated small nanoparticles which appear ordered in rows, commensurate with the graphitic steps (Figure 4a and Figure S14, Supporting Information). As these internal anchoring sites possess high local curvature and consequent bond strain, they are highly reactive toward oxidation and therefore are likely modified during the pore formation procedure (Figure 4b); this results in a consequential lack of nanoparticle organization within the porous nanotube reactors relative to that seen using as-received GNFs. However, like the as-received GNF sample, nanoparticles clearly form on defective areas which aid their adsorption, with the greatest proportion of nanoparticles found around the newly formed pore openings, suggesting the highest defect density at these sites. Interestingly, TGA of composites prepared using identical carbon weights and precursor quantities showed that higher RuNP loading (increase of 4%) could be afforded on porous GNFs relative to the as-received analogue (11.5 and 7.6%, respectively), indicating greater accessibility of molecules to the internal surfaces and a larger number of available anchoring sites because of the pore formation procedure. Nanoparticle size analysis indicated mean sizes of 1.95 and 1.93 nm within the as-received GNF and porous GNF, respectively, thus highlighting that the controlled formation of comparably small nanoparticles is afforded regardless of the presence or absence of pores in the sidewalls of the nanotube (Figure S15, Supporting Information).

**Figure 5:**
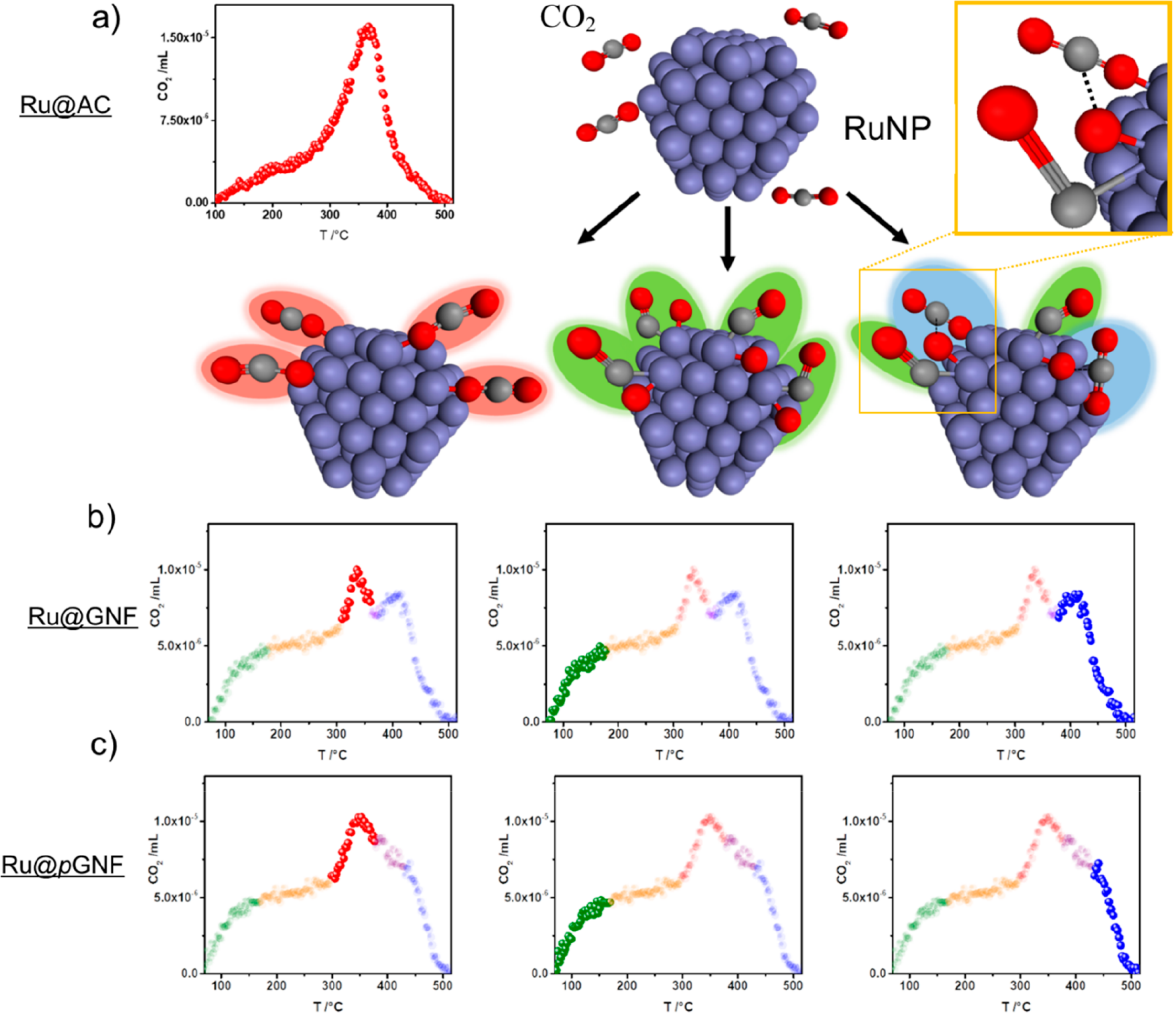
Temperature-programmed desorption profiles for CO_2_ on RuNP@AC (a), RuNP@GNF (b), and RuNP@*p*GNF (c), at a heating rate of 4 K min^–1^ with schematic representation of CO_2_ binding to Ru nanoparticles, with different modes highlighted in different colors: the process (order = 1) involving the adsorption of one CO_2_ molecule at one metal site is colored red; the process (order >1) describing the bond breaking and resulting in the formation of adsorbed CO and O is colored green; the process (order <1) where CO_2_ binds between adjacent metal sites is colored blue. The schematic yellow inset provides detailed bonding of the green and blue processes and highlights the new carbonate species formed in the nanoreactors. The latter two processes are complex and comprised of three primary processes, color coded in green, red, and blue corresponding to the proposed structural diagrams shown in the schematic diagram (transitional phases, highlighted in orange and violet, are not included in the analysis). The schematic inset provides detailed bonding of the green and blue processes.

To investigate how the defects formed in the carbon nanotubes modulate the electronic properties of the RuNPs, carbon dioxide desorption profiles of Ru nanoparticles within RuNP@*p*GNF and RuNP@GNF were examined (both with residual weight loadings of 8% Ru). CO_2_ is known to form various intermediates on metal particle surfaces that can affect the kinetics of sorption.^39,40^ Analysis of the temperature-programmed desorption (TPD) profiles by a linearization method allows the determination of the orders of sorption reactions.^41^ As the TPD of the GNF without the nanoparticles does not display any desorption process, the processes associated with the composite material must correspond to the contribution of the RuNP (Figure S16, Supporting Information). RuNP@ deposited onto amorphous carbon (RuNP@AC) were also probed, as this material is seen as an industry standard and would create benchmark conditions to test our novel support materials (Figure 5a).

The CO_2_ TPD profile for RuNP@AC indicates a single desorption process between 100 and 500 °C (Figure 5a), whereas for RuNP@GNF and RuNP@*p*GNF TPD profiles are significantly more complex, showing up to three different processes within the same temperature range. These are best observed at the slowest heating ramp of 4 °C min^–1^ (Figure 5b and c; Figure S17, Supporting Information).

To gain insight into the chemical properties and energy levels of the desorbed CO_2_, a complete analysis of the peaks was performed while individual processes were separated before the analysis (Figures S18, S19, and S20, Supporting Information). This method deduced that the CO_2_ desorption on RuNP@AC is a first-order process with an activation energy of 40.3 ± 0.5 kJ mol^–1^, which corresponds to one CO_2_ molecule binding to one surface site and represents the sole process on RuNP@AC. Similarly, first-order processes can be determined for RuNP@GNF and RuNP@*p*GNF with activation energies of 42.4 ± 1.7 and 49.9 ± 1.5 kJ mol^–1^, respectively (Table S3, Supporting Information). In addition, third-order and 0.8th-order processes have been found in RuNP@GNF and RuNP@*p*GNF, overlapping significantly which complicates the overall peak analysis.

The TPD profiles suggest that encapsulating Ru nanoparticles in GNFs alters the electronic and steric properties of the nanoparticles compared to Ru nanoparticles deposited onto amorphous carbon. The GNFs can act as an electron donor or acceptor as well as impose spatial confinement around the catalytic nanoparticles within the cavity. Desorption processes with orders greater than one strongly suggest a bond dissociation in CO_2_ (most likely resulting in the formation of CO and O adsorbed onto the surface of RuNP; Figure 5a—intermediates highlighted in green and in inset), while an order of less than one suggests CO_2_ bonding in between Ru sites (Figure 5a—intermediates highlighted in blue and in inset). Mechanistically, these observations suggest that in the first step in all cases CO_2_ is adsorbed to the Ru surface, binding one CO_2_ to one Ru atom (Figure 5A—red points in desorption profiles schematically shown as intermediates highlighted in red), followed by the C–O bond breaking and formation of CO and O adsorbed on the surface (Figure 5a—intermediates and points highlighted in green). This step might be accompanied by the formation of adsorbed carbonate by a neighboring CO_2_ adsorbed onto the surface, resulting in the desorption process with an order <1 (Figure 5A—intermediates and points highlighted in blue). This indicates that encapsulation of RuNP in GNFs allows the formation of intermediate carbonate species proposed previously in the hydrogenation of CO_2_ and the oxidation of CO.^39,40^

By simply employing the nanoreactor model, it has been shown that new surface intermediates are now accessible, and the activation energy can be increased to tune these intermediates when comparing to an industry standard like RuNP@AC (Tables S2 and S3, Supporting Information). However, this can be further modulated by introducing pores into the GNF material, which allows for the fine-tuning of the unique electronic properties already present in GNFs. These results suggest that, according to the Evans–Polyani principle, the strongest absorption of CO_2_ can be expected for RuNP@*p*GNF material followed by RuNP@GNF and finally by RuNP@AC. Selecting and tuning the nanoreactor system, therefore, allow control over the bonding of CO_2_ to the metal, an important step in designing and optimizing catalytic nanomaterials for utilizing CO_2_. It is postulated that two important factors increase the activation energy of the holey nanomaterial. First, the distorted carbon lattice alters the electronic properties of the support allowing stronger binding to catalyst nanoparticles, perturbing electron density from the RuNP structure which results in stronger adsorption of CO_2_ than in the case of the pristine carbon lattice. Second, the introduction of the pores in the GNF creates new microenvironments for catalyst nanoparticles to reside that enhance CO_2_ binding on RuNPs. It is likely that the intimate contact and increased spatial confinement at these new sites allow for maximized electronic interactions between host-nanoreactor and guest-catalyst due to multiple overlapping orbitals which can drive increased reactivity at the catalyst surface.

After temperature-programmed desorption, the porous carbon RuNP@*p*GNF nanoreactors are intact (Figure 4c) with only a small increase in nanoparticle size from 1.93 to 2.46 nm (consistent with RuNP@GNF) (Figures S15 and S21, Supporting Information), indicating that the porous nanoreactors provide effective stabilization of catalytic nanoparticles.

### Porous Carbon Nanoreactors in Liquid-Phase Reactions

**Figure 6:**
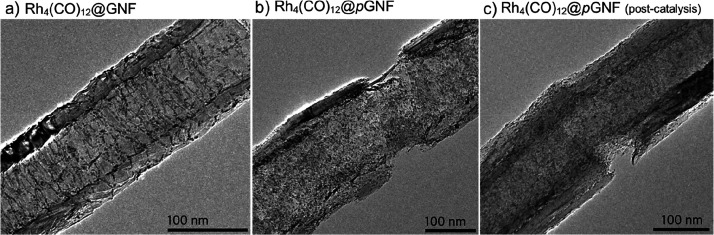
Rh_4_(CO)_12_ molecular catalysts inside pristine (a) and porous GNF (b). TEM after the hydrosilylation reaction (c) shows minimal agglomeration of the catalyst indicating effective stabilization of the molecular catalyst within *p*GNF nanoreactors.

In catalytic nanoreactors, the synergy of interactions between the nanotube, confined catalysts, and reactants increases local concentrations of reactants around catalytic centers and modulates the activity,^20,42^ selectivity,^43^ and stability^44,45^ of the catalyst, all features of which underpin the potential of carbon nanotubes for preparative-scale synthetic chemistry.^8^ Increasing the concentration of aromatic reactants within GNFs has been demonstrated previously, which not only resulted in the promoted formation of aromatic products inside carbon nanoreactors but also presented significant implications for the pathway of catalytic hydrosilylation reactions.^46^ The introduction of pores in the carbon nanoreactor is expected to increase the accessibility of the internal channel to small molecules, which may result in further modulation of reaction selectivity. Considering the affinity of carbon nanoreactors for aromatic species, we selected phenylacetylene as a reactant possessing aromaticity. A molecular catalyst Rh_4_(CO)_12_ was loaded into carbon nanoreactors using gas-phase deposition. Similarly to the RuNP discussed in the previous section, TEM imaging and EDX analysis confirmed that the catalyst resides at the internal step edges or pores in the pristine GNF and porous *p*GNF, respectively (Figure 6a and 6b and Figure S22, Supporting Information).

The reaction of phenylacetylene and triethylsilane catalyzed by rhodium yields five potential products, including three products of addition (α, β-(*Z*), and β-(*E*)) and two of dehydrogenative silylation (DS) (Table 2), with the product ratios β-(*Z*):DS and β-(*Z*):β-(*E*) useful indicators of the reaction pathway, sensitive to the local environment around catalytic centers, such as the reactant concentration (Figure S23, Supporting Information).^12,46,47^ The impact of the pores in the GNF sidewall is manifested in two effects observed for the hydrosilylation reaction. First, the decrease in β-(*Z*):DS products confirms the higher local concentration of phenylacetylene than triethylsilane within the porous nanoreactor than in GNFs without pores (Table 2). The β-(*Z*):DS ratio is sensitive to the concentration of the reagents present around the catalyst, and an excess of phenylacetylene promotes the β-H elimination step and DS products as observed in our porous nanoreactors. This observation, where the threefold increase in the local concentration of phenylacetylene inside nanoreactors resulted in the promotion of the β-H elimination pathway to consume the excess of aromatic alkyne, is consistent with our previous work.^44^ Pores in *p*GNFs appear to act as additional entry points allowing easier access of phenylacetylene into the nanoreactor, thus providing a concentration of phenylacetylene evenly heightened throughout the nanoreactor length, whereas in the case of pristine GNF—a large aspect ratio nanoreactor with only two entry points (typical length of a GNF ∼10–50 μm)—catalytic centers in the middle of the GNF would experience a lower concentration of phenylacetylene than at the ends. The second effect is shown in the decrease in β-(*Z*):β-(*E*) observed in Rh_4_(CO)_12_@*p*GNF as compared to Rh_4_(CO)_12_@GNF, indicating that in the *p*GNF nanoreactor the formation of the thermodynamic products is promoted, a consequence of the location and environment of the catalyst in *p*GNFs. From previous work, the presence of unique anchoring points in the GNF (step edges) sufficiently changes the nature of the catalyst due to the interactions between the carbon and catalyst molecules which remove destabilizing steric repulsion between adjacent Ph and SiEt_3_ and promote the β-(*E*) isomer. In this work, it was observed that these step edges have become modified because of the etching process but still provide sufficient anchoring points for catalyst molecules. The modification or opening of the carbon step edges combined with new anchoring sites at the periphery of the pores in the *p*GNF appears to provide more intimate contact between the carbon and catalyst molecules, modulating the environment around the catalytic center and promoting the selectivity of the less sterically strained β-(*E*) isomer when compared to the pristine GNF nanoreactor (Table 2). These observations demonstrated that *p*GNFs work as effective nanoreactors for liquid-phase reactions, supporting the fact that the enhanced local concentration of reactants and modulated catalytic centers offers additional mechanisms to tune the selectivity by the introduction of the pores in the walls of nanotubes.

**Table 2:**
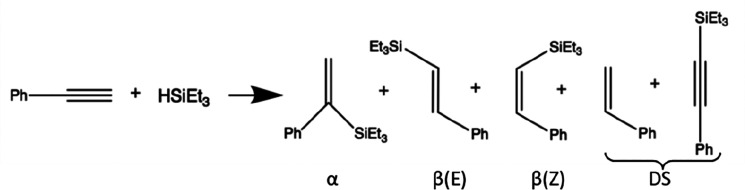
Effect of Pores in Carbon Nanoreactors on the Selectivity of Reaction of Phenylacetylene and Triethylsilane^a^

			regioselectivity	
catalyst	container	reaction duration (days)	β(*Z*):β(*E*)	β(*Z*):DS
Rh_4_(CO)_12_	GNF	6	0.8:1	1.8:1
Rh_4_(CO)_12_	*p*GNF	6	0.5:1	1.0:1
Rh_4_(CO)_12_	GNF	12	0.8:1	2.2:1
Rh_4_(CO)_12_	*p*GNF	12	0.5:1	1.4:1

a All reactions were performed at a normalized catalyst loading of 2.7 mmol % Rh_4_(CO)_12_. Conversions not exceeding 20% were deemed suitable for this reaction as it permitted evaluation of the effect of the confinement of catalyst centers in carbon nanoreactors and yielded a stable (time-independent) distribution of products on a suitable measurement time scale.

## Conclusions

Depositing chromium oxide onto the surface of carbon nanomaterials, using a gas-phase approach and a chromium acetylacetonate precursor, provides an effective way to form oxidation catalyst nanoparticles. The chromium oxide nanoparticles are formed with atomically thin carbon shells that prevent their coarsening and agglomeration, and when heated in air the nanoparticles facilitate localized etching of carbon nanotube sidewalls in a highly controlled way (a self-retarding process), leading to nanopores penetrating into the nanotube cavity while preserving the cylindrical structure of the nanotube. Porous nanotubes act as vessels for gas-phase sorption (CO_2_ sorption on Ru nanoparticles) and liquid-phase environment (alkyne hydrosilylation on Rh_4_(CO)_12_) for catalytic reactions. In both cases the presence of pores does not compromise the effectiveness of carbon nanoreactors. The sorption of CO_2_ on RuNP in the nanoreactors is stronger (as compared to RuNP on amorphous carbon) indicating an alternative binding mode with the metal centers via a carbonate species. In the liquid reaction of hydrosilylation, porous nanoreactors provide a higher local concentration of aromatic reactants around Rh catalytic centers and further promote the thermodynamic products of addition when compared to pristine nanotubes. All these demonstrate that the introduction of pores in the nanotube sidewall not only retains all advantages of nanoreactors but also allows modulation of the microenvironment around the catalytic centers. This study is the first of its kind, introducing the basic principles of the presence/absence of pores in carbon nanoreactors, and highlights how porous carbon nanotubes provide both effective stabilization of catalytic centers and good access of reactants to the internal catalyst. Therefore, they may play a crucial role in nanoreactors for processes constrained by the diffusion rate, such as electrocatalytic reactions.

## Experimental Section

### General

Standard reagents and solvents were used as purchased from Sigma-Aldrich Chemicals. Chromium acetylacetonate (97%) was purchased from Arcos Chemicals, and bis(cyclopentadienylruthenium dicarbonyl) dimer and tetrarhodium dodecacarbonyl were used as purchased from Sigma-Aldrich Chemicals. Pyrograf Products Inc. supplied the graphitized carbon nanofibers (PR19-XT-HHT) with the iron content <100 ppm. Multiwalled carbon nanotubes (purity >99.5%) were obtained from the Materials and Electrochemical Research (MER) Corporation.

A JEOL 2100F FEG-TEM with an Oxford Instruments INCA X-ray microanalysis system operating at 200 kV was used for transmission electron microscopy (TEM) and EDX measurements using a sample preparation method previously reported.^10^ An Enfinium SE system was used to measure the electron energy loss spectroscopy (EELS). A Gatan 916 high tilt tomography holder, acquiring images at 1° tilt intervals over a range of 0 to +75° with fiducial markers (Au nanoparticles), was employed to deliver the tomography analysis.

A Kratos AXIS Ultra DLD instrument was used for the X-ray photoelectron spectroscopy (XPS) measurements using parameters reported in our previous work.^22^

A TA Q500 Thermogravimetric Analyzer was used for the thermogravimetric analysis. All samples were analyzed using a platinum pan and in the presence of air. The parameters for all experiments were as follows: ramp 5 °C/min from 20 to 1000 °C with an isotherm for 10 min at 1000 °C, air flow: 60 mL/min. Thermogravimetric analysis mass spectrometry was performed using the same parameters as before, but TA Q500 was equipped with an EGA furnace with a 90 mL/min flow rate. A Hiden Analytical QGA mass spectrometer was used in Bar Scan mode using the SEM (Secondary Electron Multiplier) detector between 10 and 80 *m*/*e* with electron energy 70 eV and an emission current of 20 μA. The analysis was performed using EGA soft from Hiden Analytical.

A PANalytical X’Pert Pro diffractometer was used for the powder X-ray measurements. This was achieved using a Cu K(α) radiation source (λ = 1.5432 Å, 40 kV 40 mA) in a Bragg–Brentano geometry on a Si zero background holder. The parameters for a typical experiment were the following: step size: 0.0525°, scan speed: 0.00220°/s, start angle: 5°, stop angle: 80°, and time/step: 6080 s. High-temperature PXRD measurements were performed using an Anton Parr (HTK 1200N) high-temperature oven chamber in the air up to 450 °C.

A HORIBA LabRAM HR Raman spectrometer was used to collect the Raman spectra using a method previously described by our work.^22^

### Preparation of Chromium Oxide Graphitized Nanofiber Composite Material and Pore Formation

To remove any moisture, PR19 graphitized nanofibers were pretreated by thermal treatment (500 °C) in the air for 1 h. This temperature is below the oxidation temperature of carbon for this structure. The pretreated graphitized nanofibers (Table S1, Supporting Information) were then added to a glass ampule (ampules are always *d* = 10 mm, *L* = 12 cm unless otherwise stated) with a specific ratio of chromium acetylacetonate (Table S1, Supporting Information) and sealed under vacuum (≈5 × 10^–5^ mbar) unless otherwise stated. The ampule was heated for 2 days at 160 °C. Once sublimed, and before the ampule was opened, it was cooled rapidly for 5 min. The resulting composite was placed into a new ampule and evacuated and backfilled with Ar three times to remove any oxygen or moisture present. The ampule was filled with Ar (∼0.5 bar) prior to decomposition. To decompose the precursor, the ampules were heated for 1 h at 500 °C. The ampule and resulting material were slowly cooled for 9 h. To initiate localized oxidation of the nanotube, the composite materials were heated under the specified condition in air (Table S1, Supporting Information) and were cooled under Ar atmosphere to halt the etching process. Removal of Cr_2_O_3_ from the porous graphitized nanofibers was performed by heating the composite in concentrated nitric acid (15.8 M, 20 mL) for 1 h at reflux. Refluxing in concentrated nitric acid for 1 h was found to remove all the chromium oxide while not damaging the main carbon nanotube structure. The removal of the chromium was investigated with several different concentrations of acid and reaction times to ensure no residual chromium oxide was left. Care was taken here to avoid residual metal when the Ru/Rh nanoparticles were added for the composite nanoreactors which may affect the performance. This sample was then diluted and filtered using a PTFE membrane. To minimize the oxygen-containing groups added to the carbon because of the acid treatment, the porous nanotubes were heated to 1000 °C for 2 h and cooled slowly all under a flow of Ar. Our attempts to cut GNF into smaller “pipes” were not successful, with the cylindrical structures tending to collapse upon shortening and leading to disordered semigraphitic carbon with no accessible internal channels. Therefore, controlled etching of pores in the sidewalls is the only way to modulate the properties of these carbon nanoreactors while maintaining their tubular topology. When using carbon nanotubes with smaller diameters, all conditions were kept and have been specified (Table S1, Supporting Information).

### RuNP@GNF and Rh_4_(CO)_12_@GNF Composite Material Synthesis

The catalytic nanoreactors were synthesized using an approach similar to the preparation of chromium oxide graphitized nanofiber composite material by loading pure or porous graphitized nanofibers (60 mg) into an ampule with either bis(cyclopentadienylruthenium dicarbonyl) dimer (2.7 mg for 11.5% loading or 2.2 mg for 7.7% loading) or tetrarhodium dodecacarbonyl (4.5 mg) and sealed under vacuum (≈5 × 10^–5^ mbar). The ampule was heated to 160 or 120 °C for 1 day, respectively. Once sublimed and before the ampule was opened, the Ru precursor was slowly heated to 250 °C. Both cases involved the ampule being cooled immediately for 5 min. The molecular Rh_4_(CO)_12_ nanoreactor was now ready to use. The ruthenium precursor encapsulated in the GNF was sealed in a new ampule, evacuated, and backfilled using Ar (repeated three times) to remove any oxygen or moisture present. Before sealing, the ampule was filled with Ar (≈0.5 bar). Decomposition was initiated by heating the ampule at 500 °C for 1 h to obtain the composite material, RuNP@GNF. This was slowly cooled for 9 h.

### CO_2_ Desorption Experiments

CO_2_ desorption measurements were undertaken with a CATLAB-PCS provided by Hiden Analytical. For the desorption measurement, 15 mg of RuNP@GNF, RuNP@*p*GNF, and RuNP@AC was loaded into the CATLAB precision quartz reactor tubes equipped with 10 mg of quartz wool to hold the samples in place. The samples were then topped with an additional 10 mg of quartz wool and compressed using a precision stamp. The prepared samples were then loaded into the allocated spot in a tubular furnace.

All samples were pretreated using a constant stream of 30 mL min^–1^ of 5% H_2_ in Ar at 350 °C. Afterward, the samples were subjected to a He stream (30 mL min^–1^) and heated to 575 °C with a heating rate of 10 K min^–1^. After reaching 575 °C, the sample was cooled under He to 40 °C and subjected to a flow of 5% CO_2_ in Ar (10 mL min^–1^). After 1.5 h the CO_2_ flow was exchanged to He (30 mL min^–1^) and heated to 575 °C with a heating rate of β. During the heating ramp, desorbing species were detected with a quadrupole mass spectrometer in real time. The sample was cooled to 40 °C with a cooling rate of 10 K min^–1^ after reaching 575 °C. At 40 °C the absorption process can be repeated facilitating TPD measurements at differing heating ramps with the same sample.

The conversion factor was used in between counts, and the volume of gas was achieved using an empty tube through which 5% CO_2_ in Ar was flown with a flow rate of 5 mL min^–1^. An optimal conversion factor was found using linear regression.

### Hydrosilylation Reactions

Typical experiments were based on our previous work.^46,47^ In brief, an argon-flushed Schlenk tube was loaded with the composite materials (2.7 mmol % Rh_4_(CO)_12_), and triethylsilane (0.72 mL, 4.5 mmol, 1 equiv) and phenylacetylene were added dropwise (0.50 mL, 4.5 mmol, 1 equiv). Homogenization was achieved using bath sonication at room temperature and then stirring at 90 °C. The reaction progress was monitored by ^1^H NMR spectroscopy. Product distributions were generated by integrating the one-proton doublets of each product, which have unique shifts which were found to match known literature values (Figure S23, Supporting Information).

## Supporting Information Available

Additional material characterization, EEL spectra, XPS analysis, Raman spectra, TGA data, EDX spectra, TEM, and SEM images supporting the development of the materials; details, profiles, and linearization profiles of TPD for the CO_2_ desorption process with corresponding activation energies; additional evidence for the synthesis of the carbon nanoreactors; TEM images, EDX spectroscopy analysis, and particle size analysis with details for the hydrosilylation reaction of phenylacetylene (PDF)

## Abbreviations

GNF: graphitized carbon nanofiber
*p*GNF: porous graphitized carbon nanofiber
CNT: carbon nanotube
Cr(acac)_3_: chromium acetylacetonate
TGA: thermogravimetric analysis
TEM: transmission electron microscopy
SEM: scanning electron microscopy
EDX: energy dispersive X-ray spectroscopy
XPS: X-ray photoelectron spectroscopy
PXRD: powder X-ray diffraction
TPD: temperature-programmed desorption
RuNP: ruthenium nanoparticle.
