# Supplementary material for: Defect Etching in Carbon Nanotube Walls for Porous Carbon Nanoreactors: Implications for CO2 Sorption and the Hydrosilylation of Phenylacetylene
Source: ACS Appl Nano Mater. 2022 Feb 7;5(2):2075–86. doi: 10.1021/acsanm.1c03803 (PMC9098111; doi:10.1021/acsanm.1c03803)
Supplement: Supplementary file 1 — an1c03803_si_001.pdf [file an1c03803_si_001.pdf]

Supporting Information

Defect Etching in Carbon Nanotube Walls for Porous Carbon Nanoreactors: Implications for CO_2 Sorption and the Hydrosilylation of Phenylacetylene

Maxwell A. Astle*, [a] Andreas Weilhard*, [a] Graham A. Rance, [b] Tara M. LeMercier, [a] Craig T. Stoppiello,[b] Luke T. Norman, [a] Jesum Alves Fernandes[a] and Andrei N. Khlobystov [a, b]

*Corresponding Author emails: Maxwell.Astle@nottingham.ac.uk; Andreas.Weilhard@nottingham.ac.uk.

[a]School of Chemistry, University of Nottingham, University Park, Nottingham, NG7 2RD, United Kingdom.

[b]Nanoscale and Microscale Research Centre (nmRC), University of Nottingham, University Park, Nottingham, NG7 2RD, United Kingdom.

S1 Oxidative etching of graphitised nanofibers

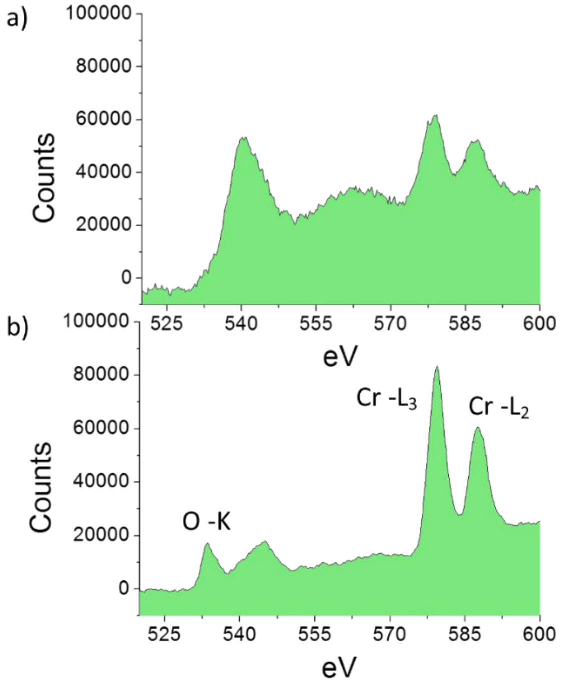

Figure S1 - EEL spectra of (a) as-synthesised (Cr_2O_3@C)/GNF and (b) Cr_2O_3 reference showing the oxygen K edge (532 eV), oxygen near-edge fine structure (544 eV) and chromium L edges (579 and 587 eV). This confirms the formation of a Cr(III) oxide in the composite material, with the broadness of the oxygen peak indicating increased oxygen vacancies in the metal oxide structure. The unusual oxygen edge in

(Cr_2O_3@C)/GNF is likely due to small chromium nanoparticles that are similar in structure to the Cr_2O_3 but may possess a higher number of oxygen vacancies.

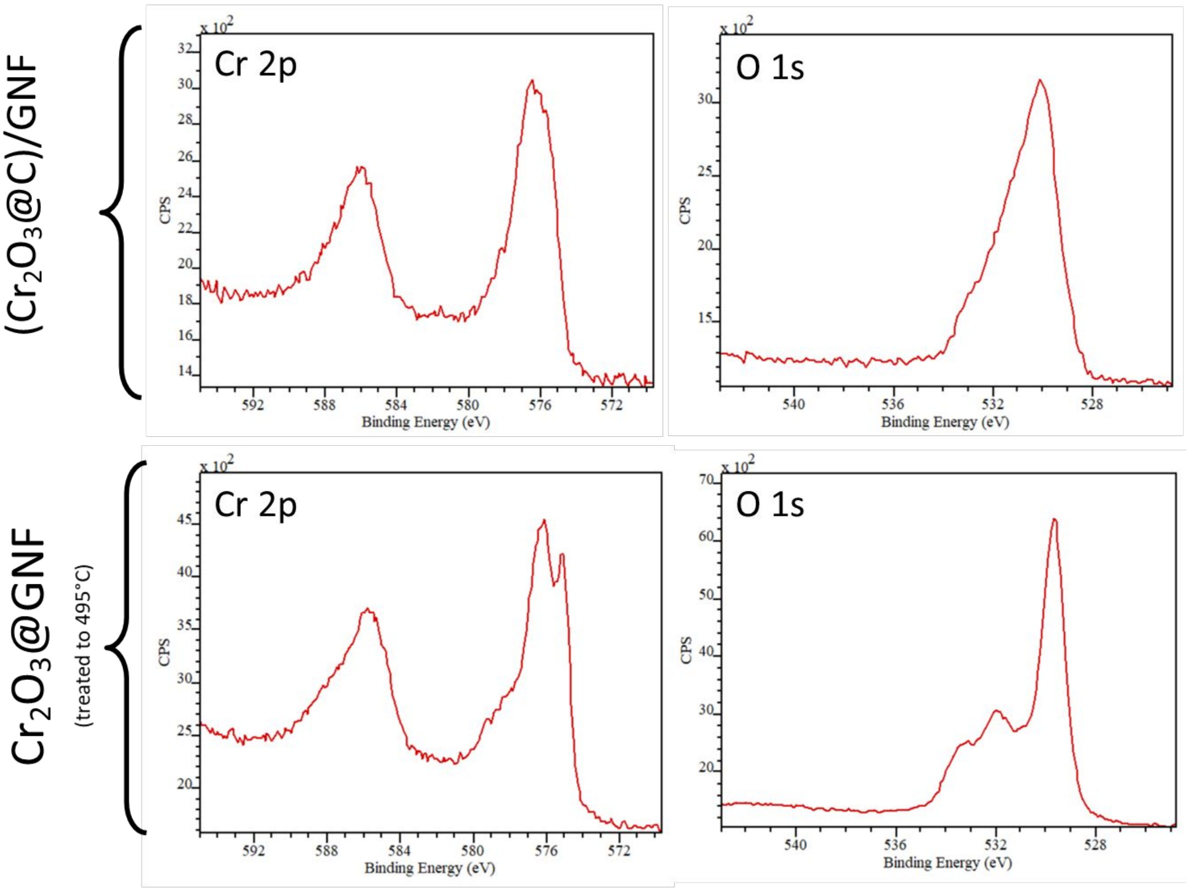

Figure S2 - XPS analysis of chromium and oxygen of (Cr_2O_3@C)/GNF before (top) and after the air treatment resulting in the etching of the carbon surface (bottom). These evidence that chromium is in the 3+ oxidation state throughout this process; the oxidised chromium being simultaneously reduced by the carbon and resulting in localised combustion of the GNF.

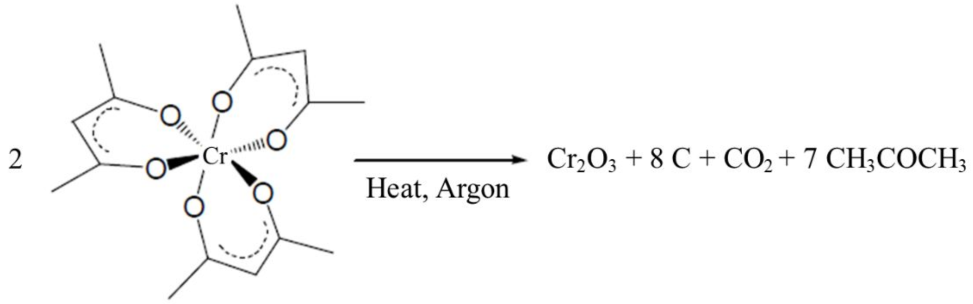

Scheme 1 – The decomposition of $Cr(acac)_3$ under an Ar atmosphere results in the formation of carbon. Although gaseous products are formed the as-produced carbon cannot be decomposed in the absence of further oxygen and therefore results in the formation of a

stabilising, carbon shell around the small chromium oxide nanoparticles (Cr_2O_3@C). Scheme produced based on results from J. Phys. Chem. **1958,** 62, **9**, 1098–110.

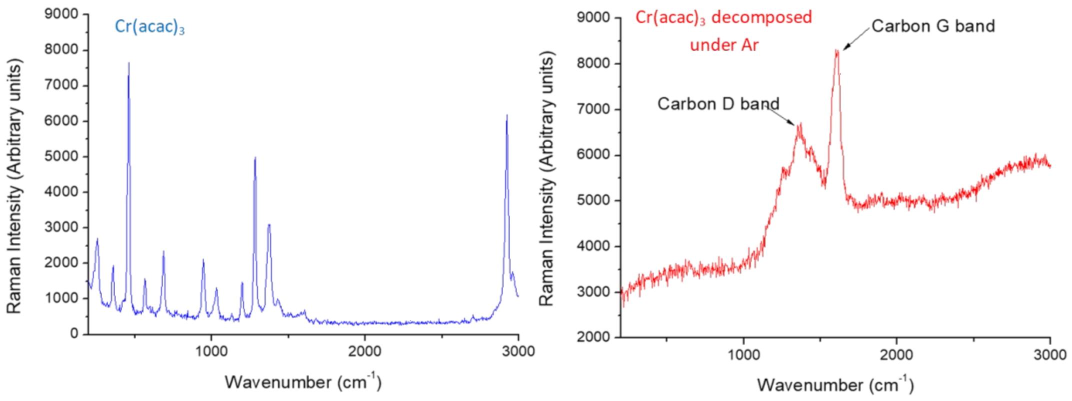

Figure S3 - Raman spectra of $Cr(acac)_3$ (blue) and the $Cr(acac)_3$ treated under the same conditions as the composite formation (red) in the absence of carbon nanotubes. This shows the formation of a carbon shell, most likely covering the small chromium oxide nanoparticles formed during decomposition of $Cr(acac)_3$. The carbon observed here is likely to be the same as that observed in (Cr_2O_3@C)/GNF, accounting for the weight loss noted in the TGA of the composite material (~330 ^{o}C), which acts as a protective layer, stabilising the small nanoparticles against undesirable further growth.

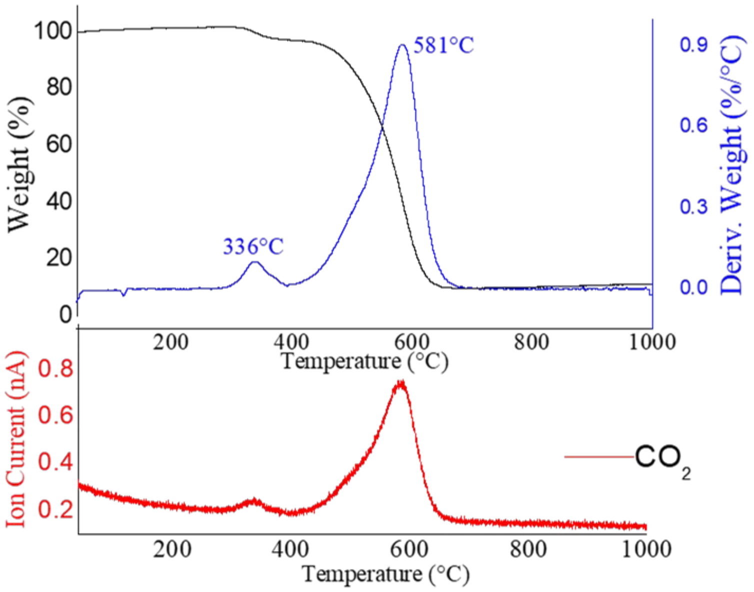

Figure S4 - Thermogram (black) and derivative weight with respect to temperature (blue) of (Cr_2O_3@C)/GNF with corresponding mass spectroscopy gas analysis (red) for CO_2 (44 m/z). This highlights that the weight loss at 336 ºC is due to the oxidation of carbon, most likely associated with less thermally stable amorphous carbon passivating the metal oxide nanoparticles, rather than the carbon of GNF itself which oxidises at a much higher temperature.

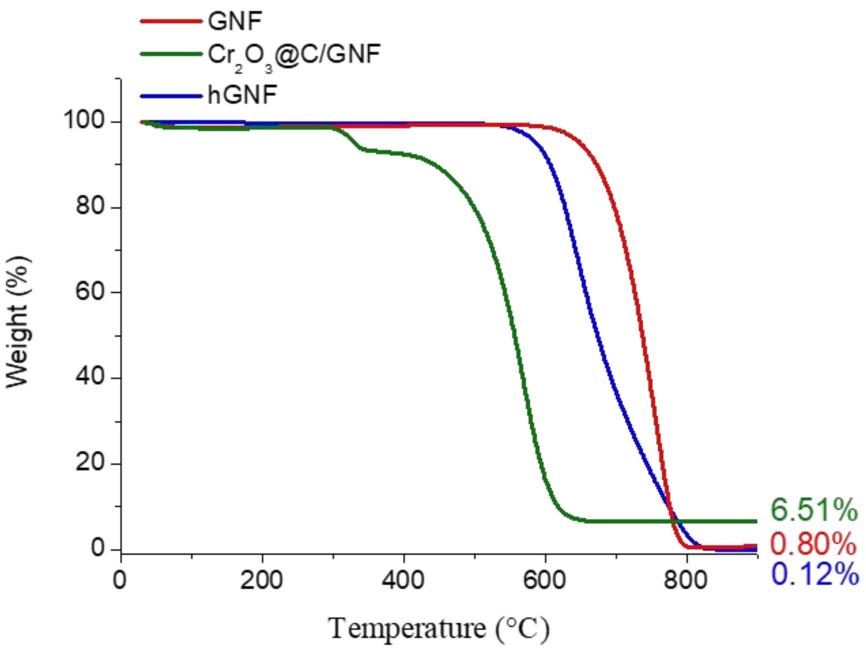

Figure S5 - TGA of GNF (red), the composite material of (Cr_2O_3@C)/GNF (green) and the final porous carbon nanoreactor after acid treatment (blue). This shows that chromium appears to have a catalytic effect in lowering the GNF combustion temperature and that the *p*GNF are more thermally stable following its removal. Due to the formation of the holes in the structure, the onset temperature of combustion of the GNF has also decreased and forms a much broader derivative consistent with the level and range of defect formation within the sample.

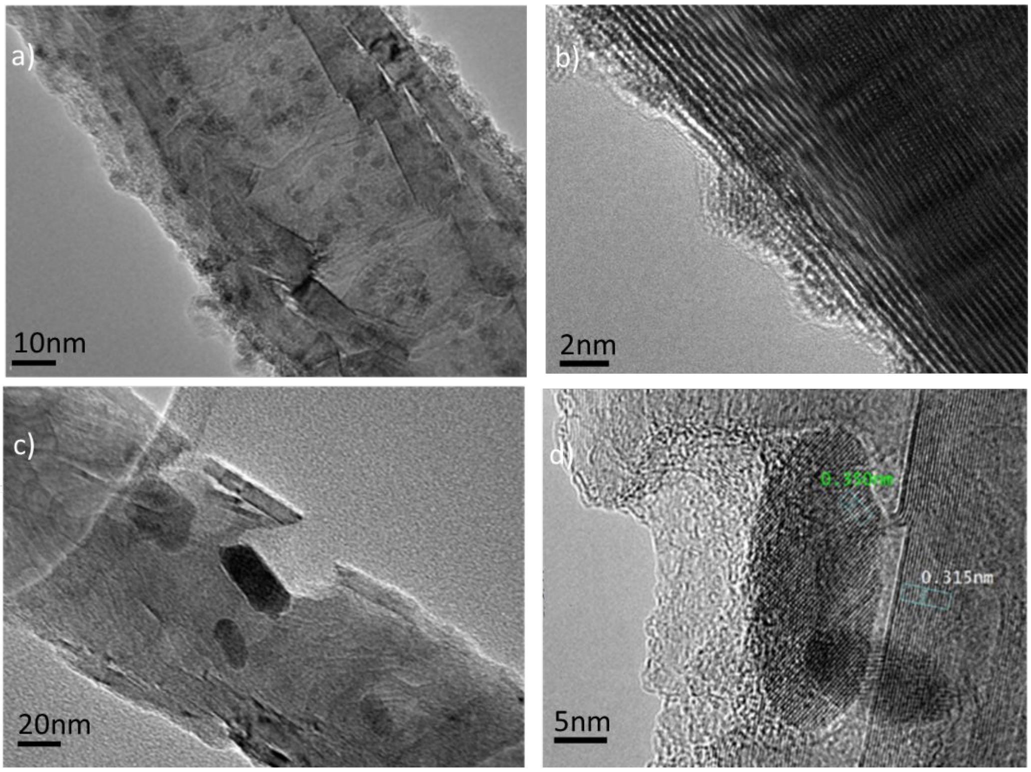

Figure S6 - TEM images of (a, b) as synthesised ($(Cr_2O_3@C)$/GNF and (c, d) Cr_2O_3@GNF after thermal treatment and weight loss at 385°C showing localised combustion of the carbon structure.

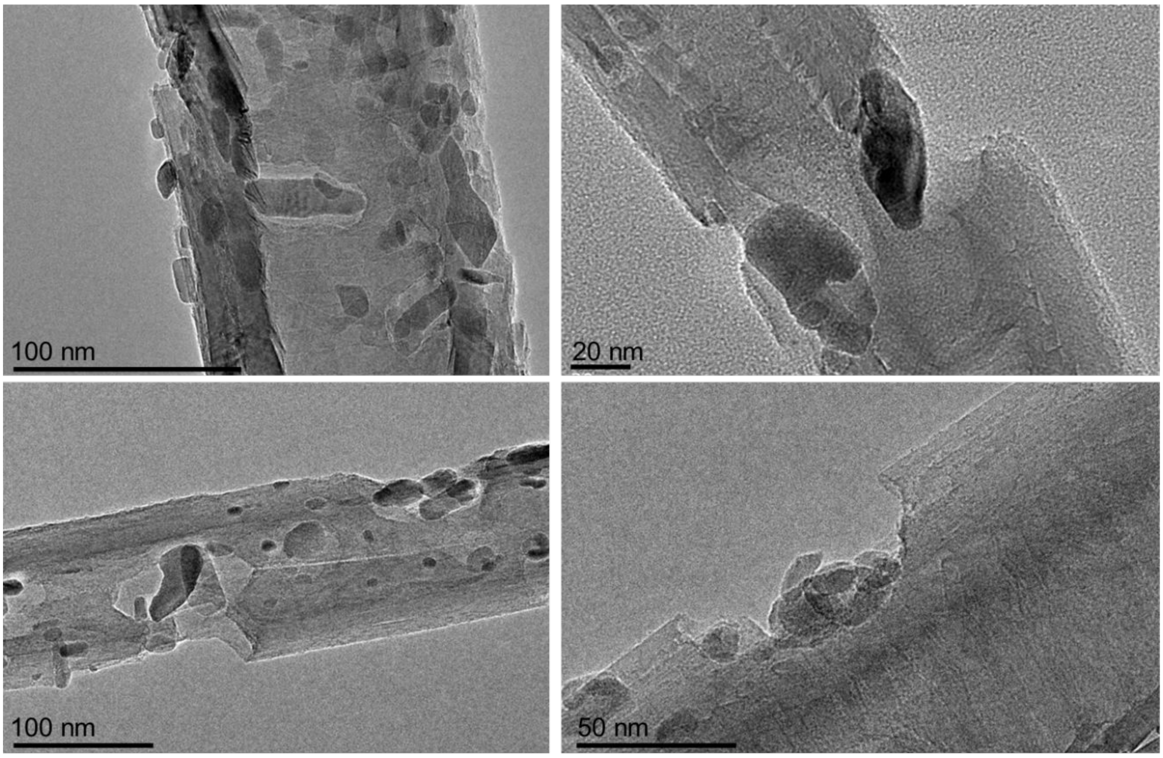

Figure S7 - TEM images of the (Cr_2O_3@C)/GNF composite material before acid treatment showing the oxidation potential of loaded chromium oxide to locally combust the carbon nanotube exterior surface and generate defects. Carbon pores and defects clearly surround the

catalytic nanoparticle which grows because of thermal treatment and oxidises the carbon at the same time due to the intimate contact between the oxide and carbon.

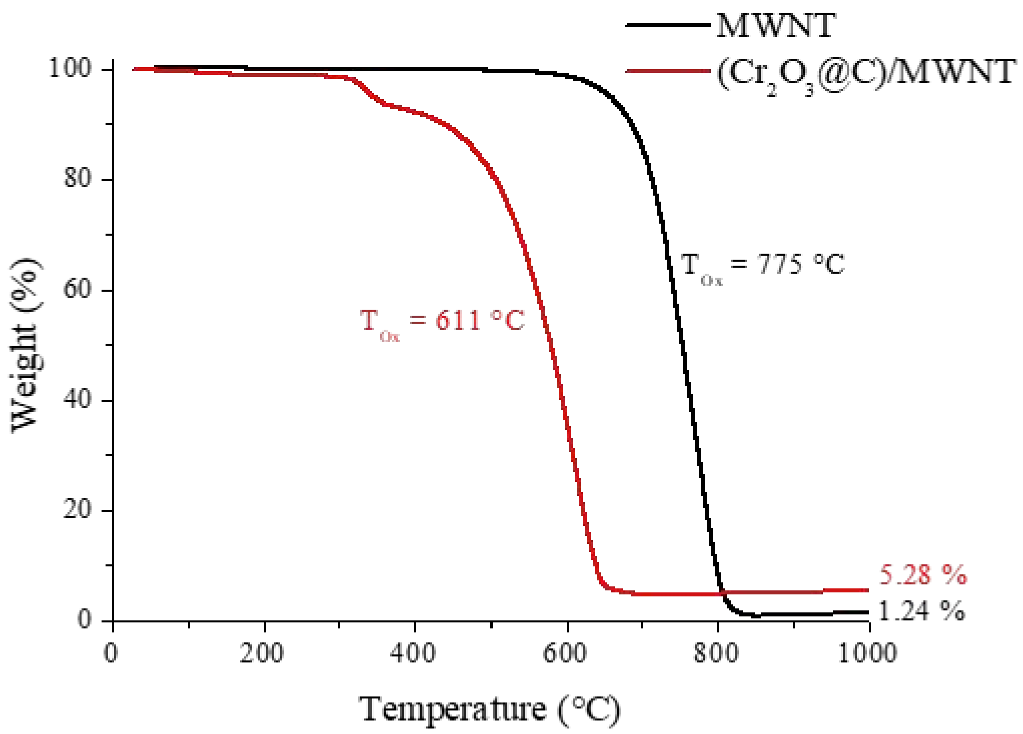

Figure S8 - Thermogravimetric analysis of hole formation in multiwalled nanotubes (MER PD30), temperature of oxidation of carbon nanotube (T_{Ox}) measured from the derivative of the TGA profile.

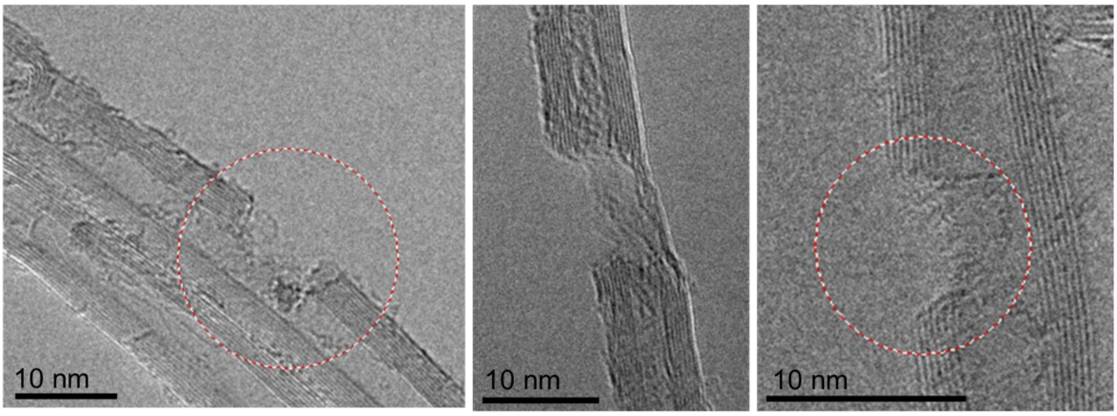

Figure S9 - TEM images of multiwalled carbon nanotubes (PD30) heated to 470 ^{o}C to generate porous carbon nanotubes.

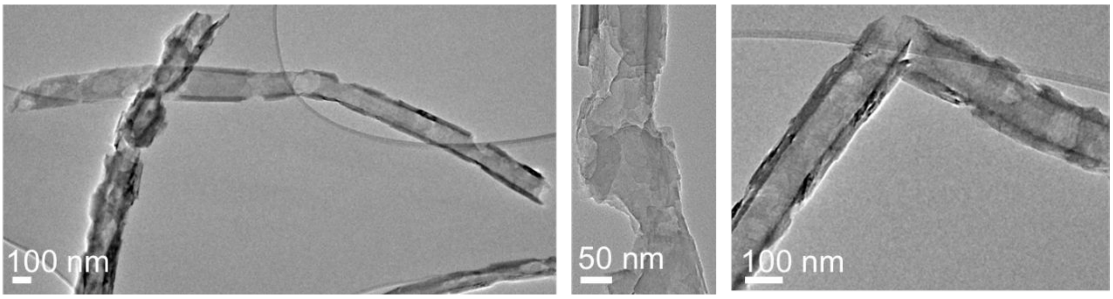

Figure S10 - TEM images graphitised nanofibers during the fast-thermal process which negatively impacted the controlled formation of pores and lead to cutting or destruction of the nanotubes.

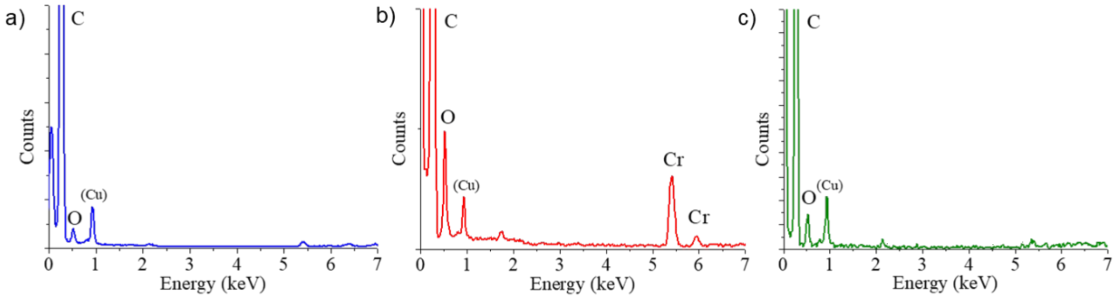

Figure S11 – EDX spectra of GNF (a), (Cr_2O_3@C)/GNF (b) and *p*GNF (c). EDX supports the TGA regarding the removal of Cr from the materials following acid washing. Small peaks in the baseline corresponding to Cr (5.41 keV), Fe (6.40 keV and 7.05 keV) and Au (2.21 keV) appear even when the pure GNF is used relating this to the steel rod and gold screws used in the TEM sample holder.

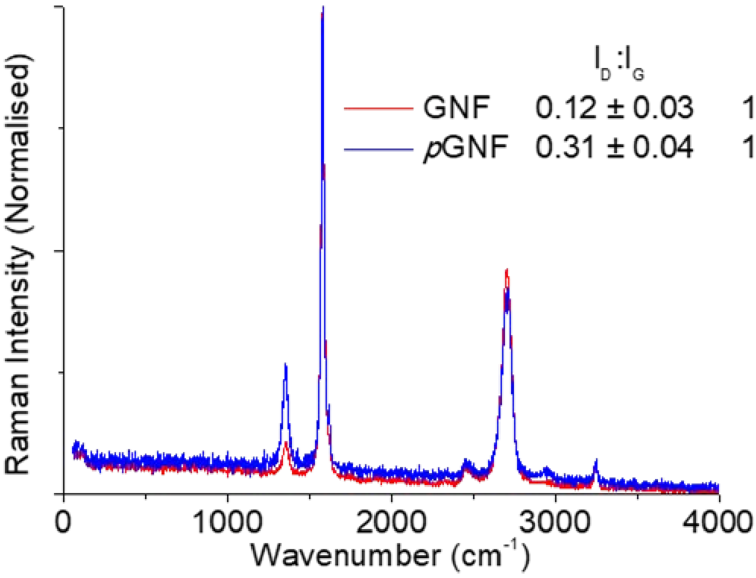

Figure S12 – Representative Raman spectra of GNF (red) and *p*GNF (blue) highlighting the increased structural disorder present after pore formation, typified by the clear increase in I_D:I_G and decrease in I_{2D}:I_G which indicate an increase in intra- and inter-layer disorder, respectively. However, we cannot really comment on the nature of the defects using these techniques due to the significant number of layers of carbon within the GNF system which makes the concentration of the defects quite small. Spectra were acquired using a 532 nm laser (at 0.3 mW power) and a 600 lines mm^{-1} rotatable diffraction grating, conferring a spectral resolution of better than 1.8 cm^{-1}.

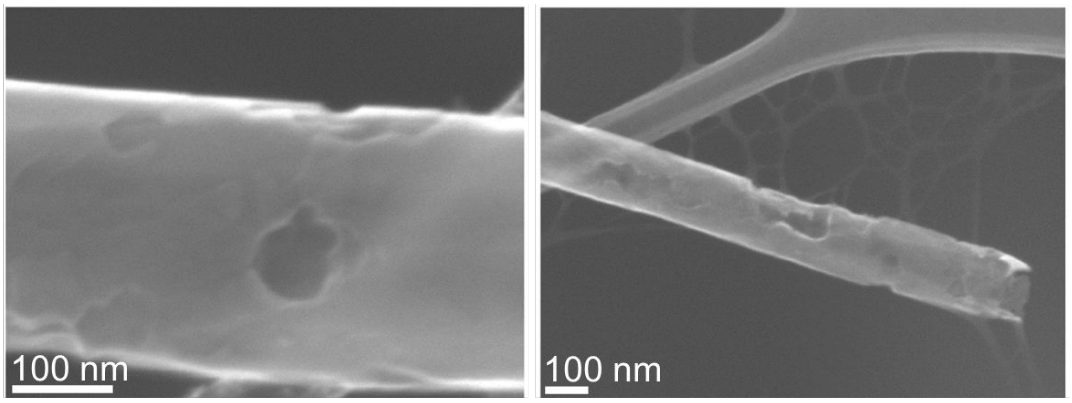

Figure S13 - SEM images of porous carbon nanotubes. Scanning electron microscopy (SEM) was performed on a Field Emission Gun (FEG) SEM operating at 15 kV.

Table S1 – Experimental condition for pore formation in carbon nanoreactors

Sample	Nanotubes used	Precursor : Nanotubes experimental conditions (mg)	µmol of precursor to 1mg of nanotubes	Pore formation conditions [a]			
				Heating equipment used	Ramp Rate (^{O}C/min)	Hold Temperature (^{O}C)	Isotherm (minutes) [b]
A	GNF	18 : 50	1.02	TGA	10	375	10
B	GNF	18 : 50	1.02	TGA	10	495	10
C	GNF	12 : 50	0.68	TGA	10	495	10
D	GNF	6 : 50	0.34	TGA	10	495	10
E	GNF	18 : 50	1.02	TGA	15	495	10
F	GNF	18 : 50	0.68	TGA	5	495	10
G	GNF	12 : 50	0.68	Tube furnace	10	495	10
H	GNF	12 : 50	0.68	Tube furnace	10	495	8
I	GNF	12 : 50	0.68	Tube furnace	10	495	5
J	MWNT (MER)	18 : 50	1.02	TGA	10	495	10

[a)] Ramp rates were all performed under air. Thermal treatments for samples A-F are all performed using small batches in a thermogravimetric analyser which allowed for confident control of the parameters. The thermal treatments for G-i represent a scaled-up procedure which was performed in a tube furnace where gas flows and ramp rates were less controllable. [b)] Once isotherms were completed, samples were cooled in an Ar atmosphere.

S2 Porous carbon nanoreactors in gas-phase reactions

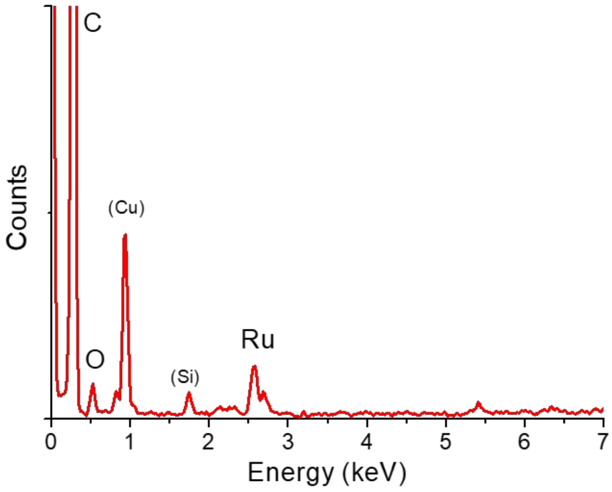

Figure S14 - EDX spectrum of Ru@GNF.

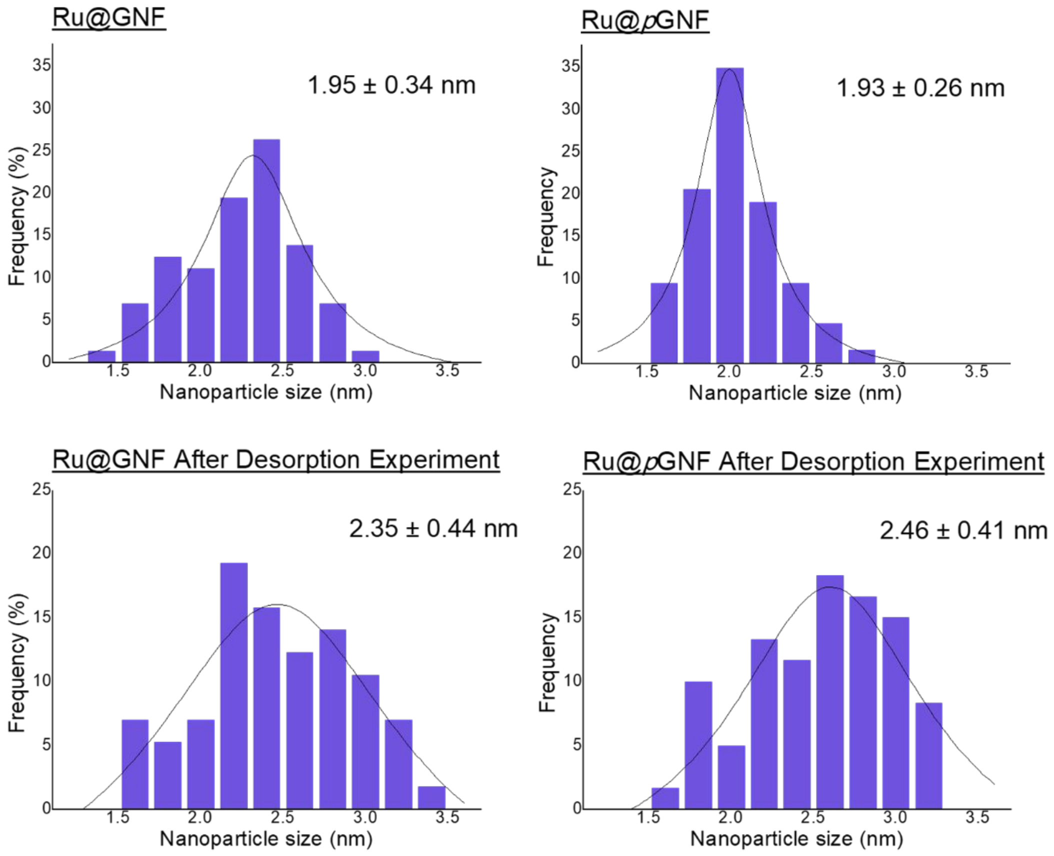

Figure S15 - Nanoparticle size distribution of RuNP on GNF and pGNF before and after the heating CO_2 desorption procedure. The nanoparticle size profiles appear similar regardless of the presence/absence of holes in the materials, and there is small increase in nanoparticle size after the desorption experiments.

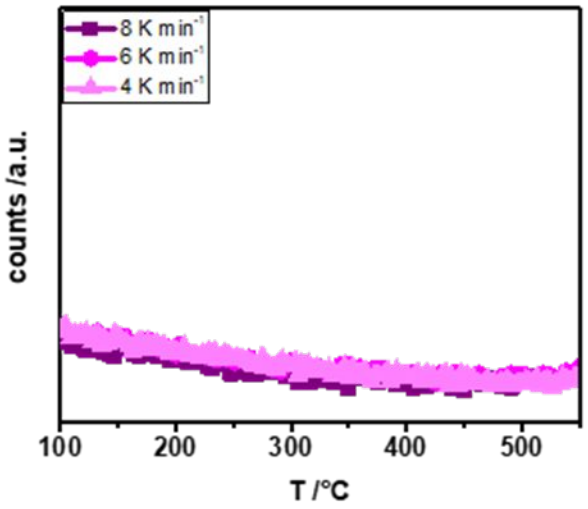

Figure S16 - TPD profiles of CO_2 with 15 mg of pure GNF at β=8 K min^{-1}, β=6 K min^{-1} and β=4 K min^{-1}. As pure GNF do not display any CO2 desorption processes, any desorption process observed in the following must be associated with Ru NP in the bespoke materials.

The conversion factor in between counts and volume of gas is achieved using an empty tube through which 5% CO_2 in Ar is flown with a flow rate of 5 mL min^{-1}. An optimal conversion factor is found using a linear regression. The figures below are desorption profiles of Ru@AC, Ru@GNF and Ru@*p*GNF depicted.

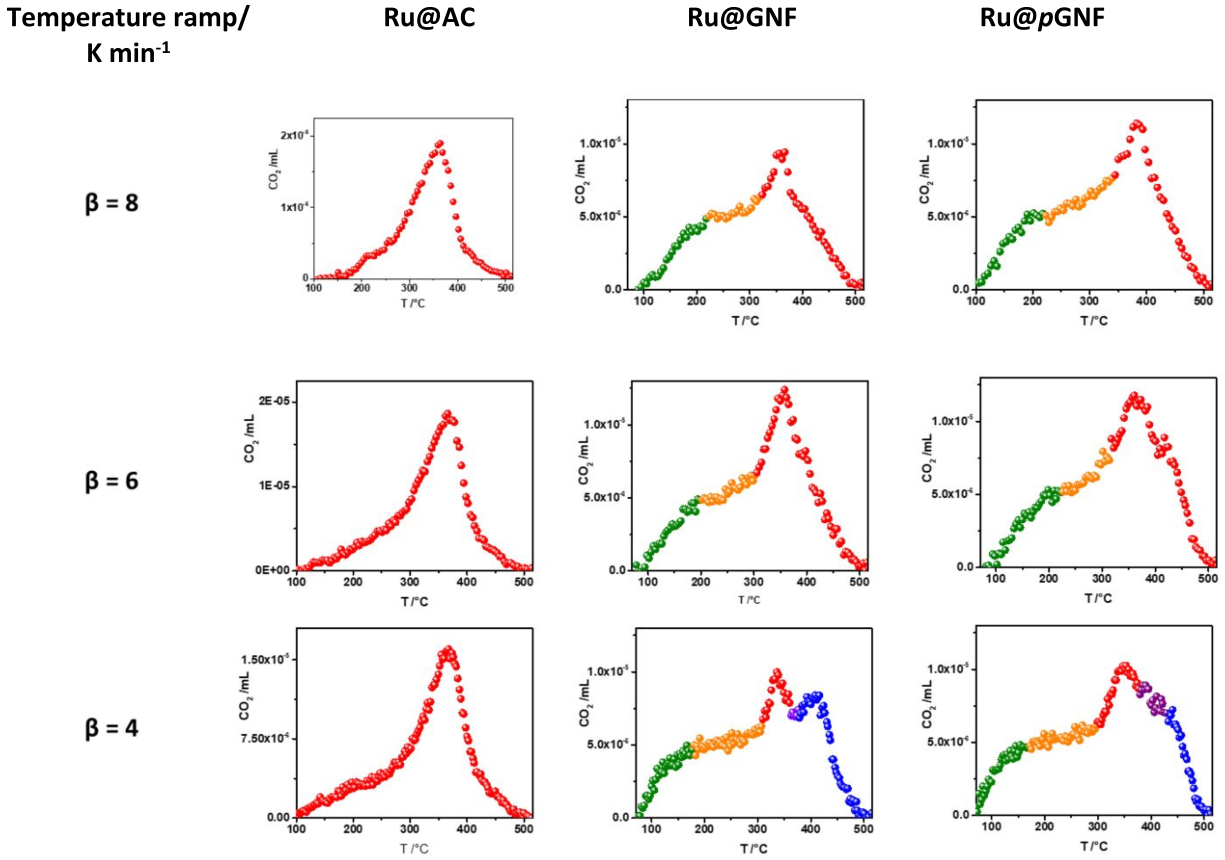

Figure S17 - TPD profiles of CO_2 with 15 mg Ru@AC, 15 mg Ru@GNF and 15 mg Ru@pGNF at β=8 K min^{-1}, β=6 K min^{-1} and β=4 K min^{-1}. Note that differing parts of the TPD profiles are colour coded solely for visualisation purposes. It is furthermore important to note that no other desorption species are observed. Analysis of the TPD profiles is achieved using complete analysis proposed by Parker et al.[1] In this fashion linearisation for the TPD profiles can be obtained using the correct orders. The linear profiles for the individual materials are depicted below.

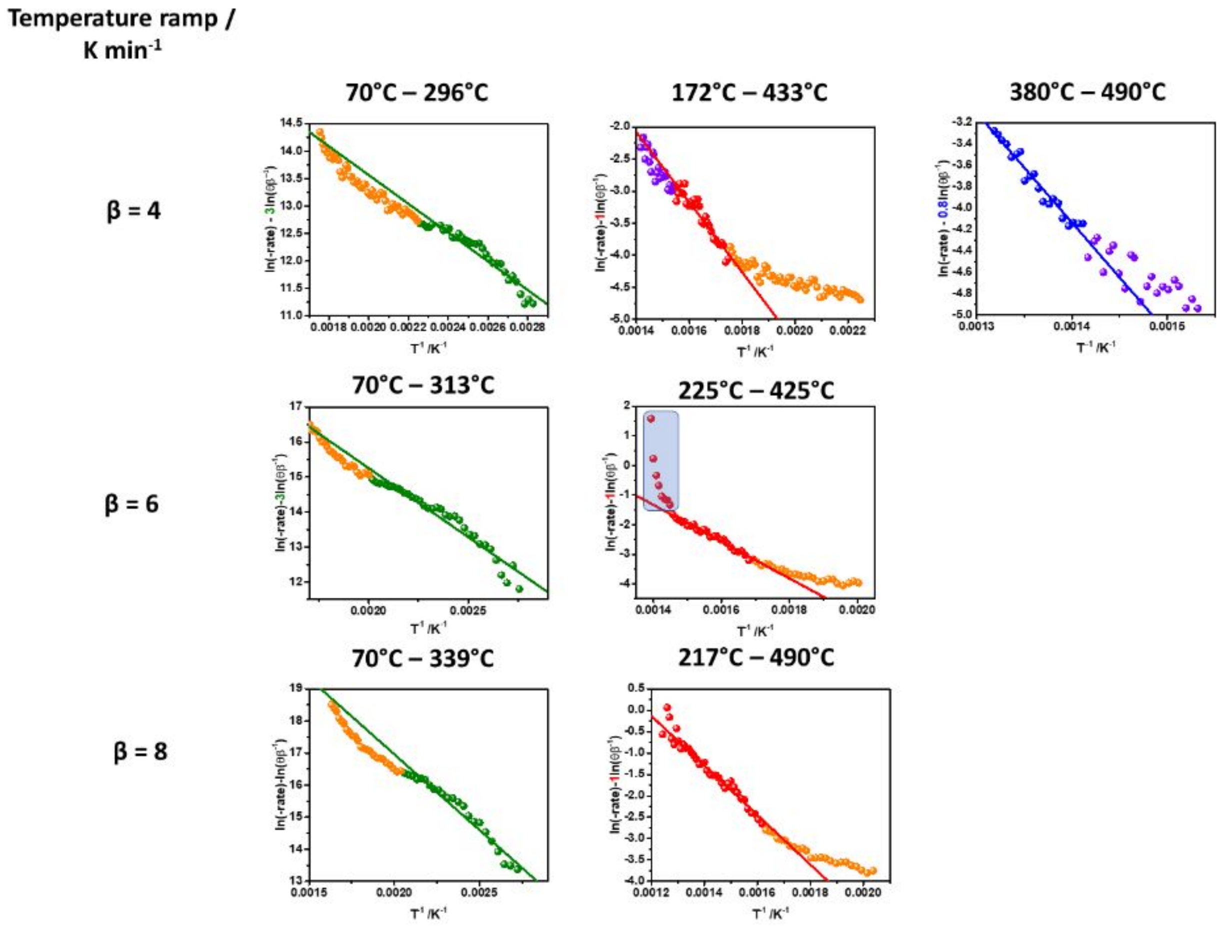

Figure S18 - Linearisation of the TPD profiles depicted in figure S17 for Ru@pGNF. The colour codes match the colour codes depicted in Figure S17.

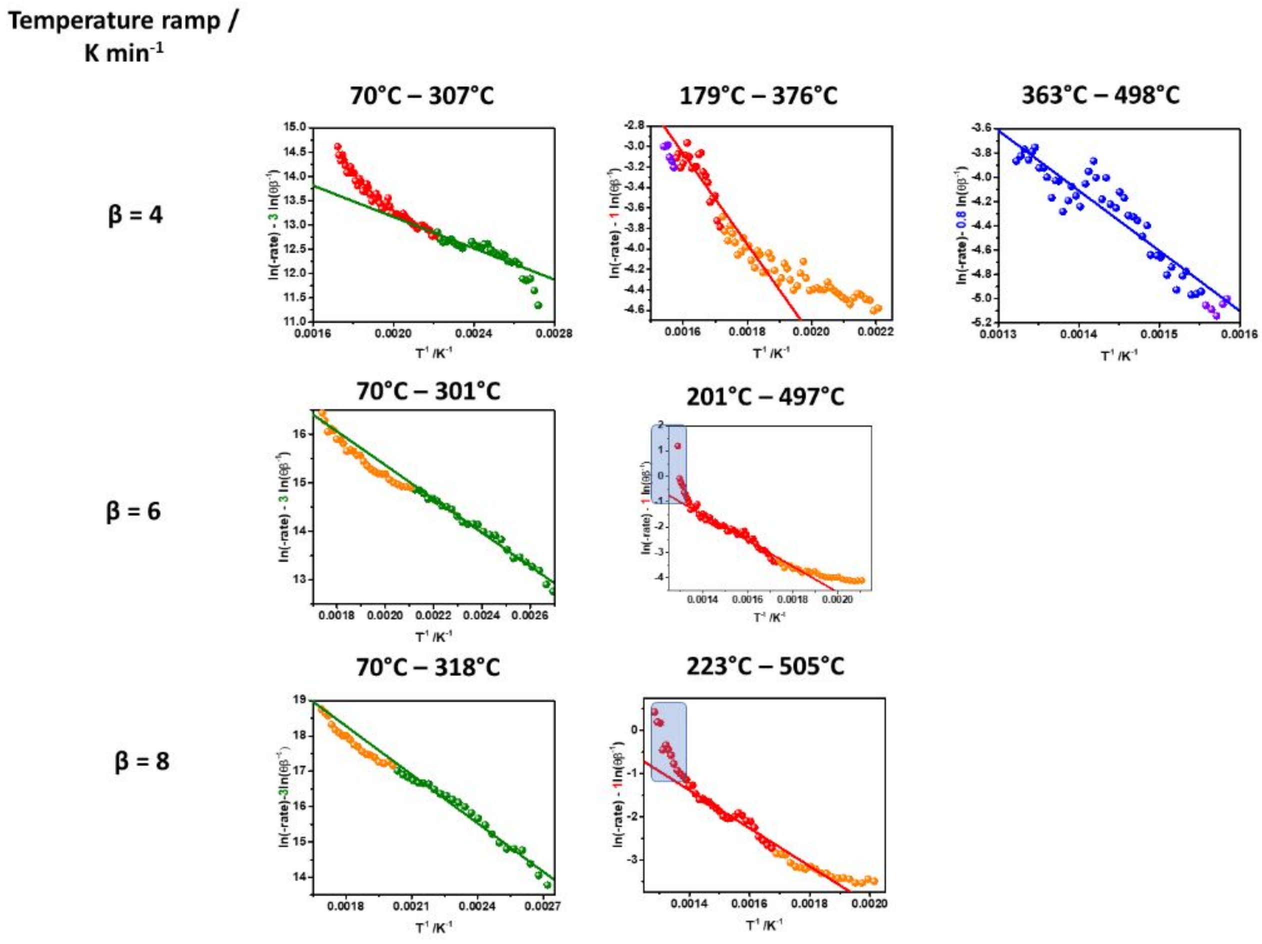

Figure S19 - Linearisation of the TPD profiles depicted in figure S17 for Ru@GNF. The colour codes match the colour codes depicted in Figure S17.

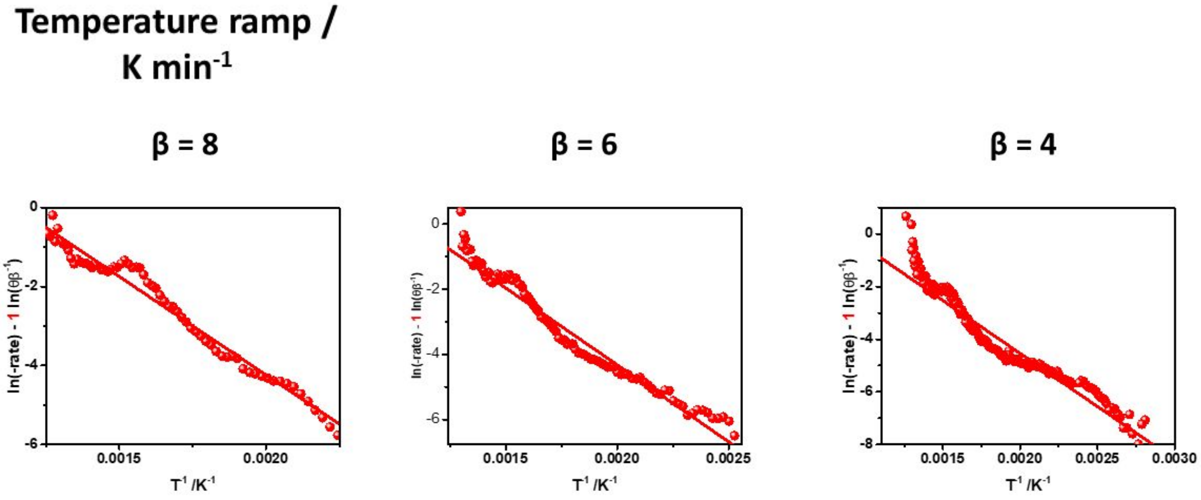

Figure S20 - Linearisation of the TPD profiles depicted in figure S17 for Ru@AC. The colour code matches the colour code depicted in Figure S17.

The tables below summarise the activation energies found for the individual desorption processes with their corresponding orders and R^2 values. In bold are values highlighted which are used for analysis. Here the highest R^2 values are found

Table S2 - Activation energies found for Ru@pGNF, Ru@GNF and Ru@AC. Green depicts the third order process, blue the 0.8th order process and red the 1st order process.

Entry	Material	Beta / K min^{-1}	E_a 3rd order process/ kJ mol^{-1} (R^2)	E_a 1st order process/ kJ mol^{-1} (R^2)	E_a 0.8th order process/ kJ mol^{-1} (R^2)
1	Ru@*p*GNF	8	**32.8 ± 1.5 (0.96)**	**48.2 ± 1.3 (0.98)**	n.a.
2	Ru@*p*GNF	6	**32.7 ± 1.4 (0.94)**	**51.6 ± 1.6 (0.98)**	n.a.
3	Ru@*p*GNF	4	21.8 ± 1.2 (0.91)	45.4 ± 2.6 (0.91)	**85.3 ± 4.4 (0.95)**
4	Ru@GNF	8	**38.2 ± 1.2 (0.98)**	36.6 ± 2.5 (0.91)	n.a.
5	Ru@GNF	6	**28.8 ± 0.7 (0.98)**	**42.4 ± 1.7 (0.95)**	n.a.
6	Ru@GNF	4	13.4 ± 1.4 (0.78)	37.0 ± 1.7 (0.66)	**41.1 ± 2.7 (0.85)**
7	Ru@AC	8	n.a.	**41.4 ± 0.9 (0.96)**	n.a.
8	Ru@AC	6	n.a.	**39.2 ± 0.9 (0.96)**	n.a.
9	Ru@AC	4	n.a.	33.4 ± 0.9 (0.92)	n.a.

The nanotube nanoreactors are permanently open (at both termini) and therefore the addition of pores in their sidewalls does not significantly increase their surface area. However, the local accessibility to catalyst active sites is clearly increased for pGNF material, as a result of the new side wall pores ensuring that access/egress of reactants/products to/from the internal channel does not have to be exclusively through the open tips (as is the case for GNF). Furthermore, we see important differences in electronic properties comparing RuNP@*p*GNF and RuNP@GNF. Thus, we demonstrate that there are two important factors that increase the activation energy of the *p*GNF: (i) the distorted carbon lattice which alters the electronic properties of the support allowing stronger binding to catalyst nanoparticles, and (ii) the introduction of the pores in GNF creates new microenvironments for catalyst nanoparticles to reside that enhance CO_2 binding on RuNP.

Table S3 - Activation energies determined for the first order desorption process for all samples.

Entry	Material	1st order process/ kJ mol^{-1}
1	Ru@AC	40.3 ± 0.5
2	Ru@GNF	42.4 ± 1.7
3	Ru@*p*GNF	49.9 ± 1.5

Table S4 - Averaged activation energies for Ru@AC, Ru@GNF and Ru@pGNF

Entry	Material	0.8th order process/ kJ mol^{-1}	2nd order process/ kJ mol^{-1}
1	Ru@AC		
2	Ru@GNF	41.1	33.5
3	Ru@*p*GNF	85.3	32.7

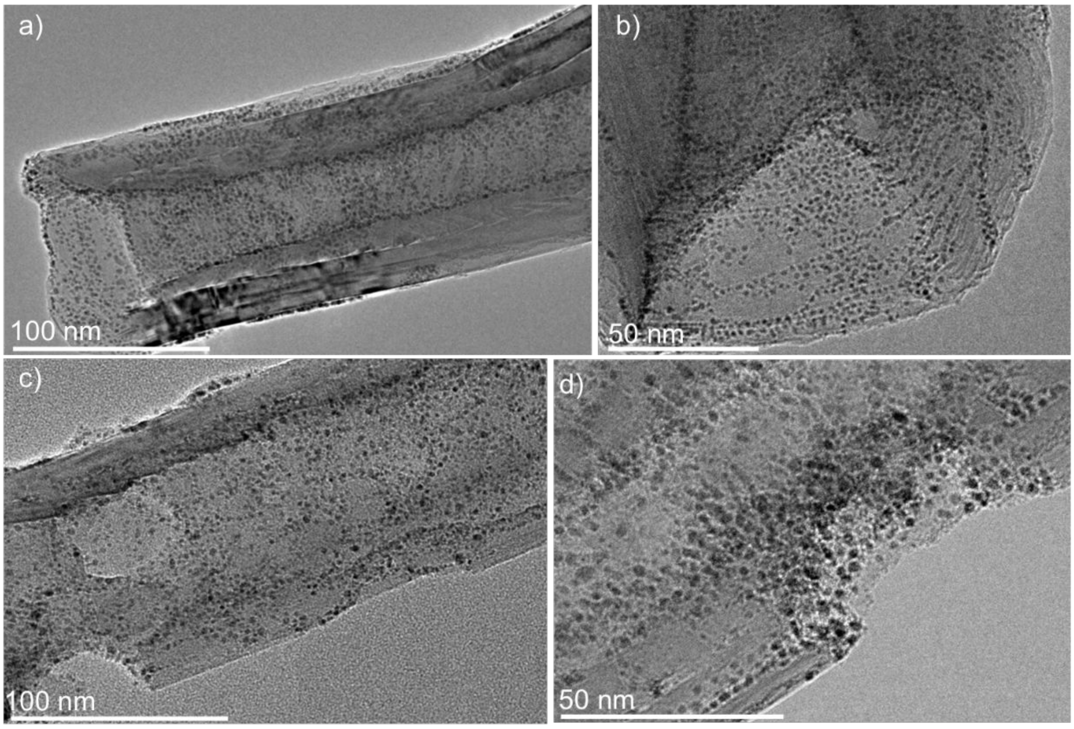

Figure S21 - TEM of RuNP loaded onto GNF (a & b) and porous nanoreactors (c & d) both after adsorption and desorption of CO_2 procedure. Small increase in nanoparticles size from 1.95 nm to 2.35 nm for the RuNP@GNF and 1.93 nm to 2.46 nm for the RuNP@pGNF associated to the high temperature conditions used during the desorption process. It is clear that both nanoreactors seem to be able to stabilise the small nanoparticles and prevent unprecedented growth of the nanoparticles.

S3 Porous carbon nanoreactors in liquid-phase reactions

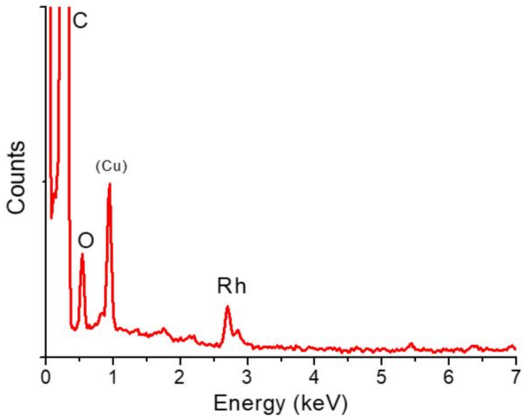

Figure S22 - EDX spectrum of $Rh_4(CO)_{12}$@GNF.

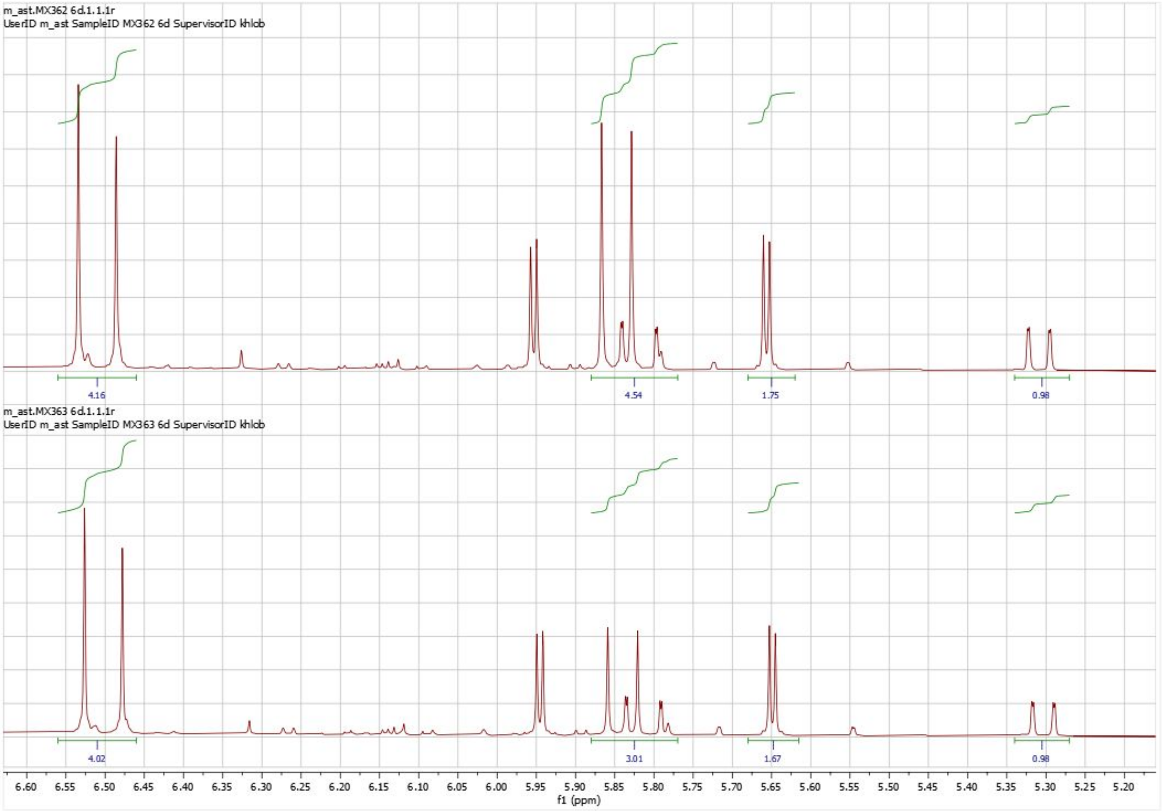

Figure S23 – ^{1}H NMR spectroscopy analysis of the crude reaction mixture following the hydrosilylation reaction of phenylacetylene and triethylsilane catalysed by molecular $Rh_4(CO)_{12}$ loaded onto GNF (top spectra) and porous GNF (bottom spectra). Standard conditions: phenylacetylene (4.5 mmol), triethylsilane (4.5 mmol), 90 ^{O}C, 6 days. Although the kinetics were sluggish with conversion not exceeding 20 %, [$Rh_4(CO)_{12}$] was deemed a suitable catalyst for this reaction – critically permitting evaluation of the effect of confinement of catalyst centres in carbon nanoreactors – as it yielded a stable (time-independent) distribution of products on a suitable measurement timescale.

References

1. Parker, D. H.; Jones, M. E.; Koel, B. E., Determination of the reaction order and activation energy for desorption kinetics using TPD spectra: Application to D2 desorption from Ag(111). *Surf. Sci.* **1990,** *233* (1-2), 65-74.
